# Strategies to decrease inequalities in cancer therapeutics, care and prevention: Proceedings on a conference organized by the Pontifical Academy of Sciences and the European Academy of Cancer Sciences, Vatican City, February 23–24, 2023

**DOI:** 10.1002/1878-0261.13575

**Published:** 2024-01-22

**Authors:** Ulrik Ringborg, Joachim von Braun, Julio Celis, Michael Baumann, Anton Berns, Alexander Eggermont, Edith Heard, Manuel Heitor, Mammen Chandy, Chien‐Jen Chen, Alberto Costa, Francesco De Lorenzo, Edward M. De Robertis, Frederick Charles Dubee, Ingemar Ernberg, Mariya Gabriel, Åslaug Helland, Rui Henrique, Bengt Jönsson, Olli Kallioniemi, Jan Korbel, Mechthild Krause, Douglas R. Lowy, Olivier Michielin, Peter Nagy, Simon Oberst, Vincenzo Paglia, M. Iqbal Parker, Kevin Ryan, Charles L. Sawyers, Joachim Schüz, Katherine Silkaitis, Eric Solary, David Thomas, Peter Turkson, Elisabete Weiderpass, Huanming Yang

**Affiliations:** ^1^ European Academy of Cancer Sciences Stockholm Sweden; ^2^ Cancer Center Karolinska Karolinska University Hospital Stockholm Sweden; ^3^ Pontifical Academy of Sciences Vatican City Italy; ^4^ Bonn University Germany; ^5^ Danish Cancer Institute Copenhagen Denmark; ^6^ German Cancer Research Center Heidelberg Germany; ^7^ The Netherlands Cancer Institute Amsterdam The Netherlands; ^8^ University Medical Center Utrecht & Princess Máxima Center for Pediatric Oncology The Netherlands; ^9^ European Molecular Biology Laboratory Heidelberg Germany; ^10^ Centre for Innovation, Tech. & Policy Research, IN+@IS Tecnico University of Lisbon Portugal; ^11^ Tata Medical Center Kolkata India; ^12^ Christian Medical College and Hospital Vellore India; ^13^ Academia Sinica Taipei Taiwan; ^14^ European Commission, Cabinet of Commissioner Stella Kyriakides Brussels Belgium; ^15^ European Cancer Patient Coalition Brussels Belgium; ^16^ University of California Los Angeles CA USA; ^17^ Beijing Genomics Institute (BGI) Helsinki Finland; ^18^ Karolinska Institutet Stockholm Sweden; ^19^ European Commissioner for Innovation, Research, Culture, Education and Youth Brussels Belgium; ^20^ Division for Cancer Medicine Oslo University Hospital Norway; ^21^ Porto Comprehensive Cancer Center Raquel Seruca (Porto.CCC Raquel Seruca) Portugal; ^22^ Stockholm School of Economics Sweden; ^23^ Science for Life Laboratory Stockholm Sweden; ^24^ Carl Gustav Carus University Hospital and Faculty of Medicine, TU Dresden Germany; ^25^ National Cancer Institute Bethesda MD USA; ^26^ CHUV Centre Hospitalier Universitaire Vaudois Lausanne Switzerland; ^27^ Department of Molecular Immunology and Toxicology and the National Tumor Biology Laboratory National Institute of Oncology Budapest Hungary; ^28^ Department of Anatomy and Histology, HUN‐REN–UVMB Laboratory of Redox Biology Research Group University of Veterinary Medicine Budapest Hungary; ^29^ Chemistry Institute University of Debrecen Hungary; ^30^ Organisation of European Cancer Institutes Brussels Belgium; ^31^ Pontifical Academy for Life Rome Italy; ^32^ University of Cape Town and African Academy of Sciences South Africa; ^33^ Cancer Research UK Scotland Institute Glasgow UK; ^34^ Memorial Sloan Kettering Cancer Center New York NY USA; ^35^ International Agency for Research on Cancer Lyon France; ^36^ World Health Organisation Lyon France; ^37^ Gustave Roussy Cancer Campus Grand Paris Villejuif France; ^38^ Garvan Institute of Medical Research The Kinghorn Cancer Centre Sydney Australia; ^39^ Beijing Genomics Institute (BGI) Shenzhen China

**Keywords:** cancer prevention, cancer therapeutics/care, healthcare, inequalities, science policy, translational cancer research

## Abstract

Analyses of inequalities related to prevention and cancer therapeutics/care show disparities between countries with different economic standing, and within countries with high Gross Domestic Product. The development of basic technological and biological research provides clinical and prevention opportunities that make their implementation into healthcare systems more complex, mainly due to the growth of Personalized/Precision Cancer Medicine (PCM). Initiatives like the USA‐Cancer Moonshot and the EU‐Mission on Cancer and Europe's Beating Cancer Plan are initiated to boost cancer prevention and therapeutics/care innovation and to mitigate present inequalities. The conference organized by the Pontifical Academy of Sciences in collaboration with the European Academy of Cancer Sciences discussed the inequality problem, dependent on the economic status of a country, the increasing demands for infrastructure supportive of innovative research and its implementation in healthcare and prevention programs. Establishing translational research defined as a coherent cancer research continuum is still a challenge. Research has to cover the entire continuum from basic to outcomes research for clinical and prevention modalities. Comprehensive Cancer Centres (CCCs) are of critical importance for integrating research innovations to preclinical and clinical research, as for ensuring state‐of‐the‐art patient care within healthcare systems. International collaborative networks between CCCs are necessary to reach the critical mass of infrastructures and patients for PCM research, and for introducing prevention modalities and new treatments effectively. Outcomes and health economics research are required to assess the cost‐effectiveness of new interventions, currently a missing element in the research portfolio. Data sharing and critical mass are essential for innovative research to develop PCM. Despite advances in cancer research, cancer incidence and prevalence is growing. Making cancer research infrastructures accessible for all patients, considering the increasing inequalities, requires science policy actions incentivizing research aimed at prevention and cancer therapeutics/care with an increased focus on patients' needs and cost‐effective healthcare.

## Graphical abstract

Here we summarize the conference proceedings of the second meeting on Cancer organized by the Pontifical Academy of Sciences together with the European Academy of Cancer Sciences. Despite the growing advances in cancer research, cancer incidence and prevalence are on the rise especially in countries with poor economic standing, which leads to a global inequality problem. The increasing demands for infrastructure support in innovative research and implementation of research outcomes in healthcare and prevention programs were discussed at the meeting. Establishing infrastructures in translational research that are sustainable and accessible for all patients is crucial in addressing inequalities in cancer treatment.

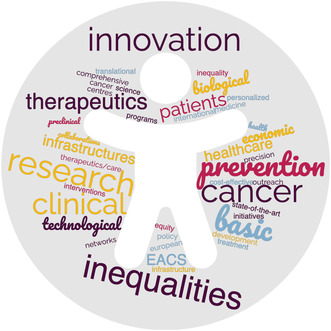

**Abbreviations**
AACR: American Association for Cancer Research
AI: Artificial Intelligence
BMBF: Federal Ministry of Education
CCC: Comprehensive Cancer Centre
CCE: Cancer Core Europe
CEE: Central and Eastern Europe
DDHMs: Data‐Driven Hall Marks
DCI: Danish Cancer Institute
DKFZ: German Cancer Research Center
DKTK: German Cancer Consortium
EACS: European Academy of Cancer Sciences
EBCP: Europe's Beating Cancer Plan
EC: European Commission
ECPC: European Cancer Patient Coalition
EMBL: European Molecular Biology Laboratory
EU: European Union
GDP: Gross National Product
GDPR: General Data Protection Regulation
HDI: Human Development Index
HICs: High‐Income Countries
HLA: Human Leukocyte Antigen
HPV: Human Papilloma Virus
HTA: Health Technology Assessment
IARC: International Agency for Research on Cancer
LMICs: Low‐ and Middle‐Income Countries
MoC: Mission on Cancer
MS: Member States
MVBs: Multivesicular Bodies
NCT: National Center for Tumor Diseases
OECI: Organisation of European Cancer Institutes
PAS: Pontifical Academy of Sciences
PCM: Precision/Personalized Cancer Medicine
RRF: Recovery and Resilience Fund
UNCAN: UNderstand CANcer

## 1 Introduction

Inequalities are systemic and often entrenched in socioeconomic and political structures within and across countries. In recent years, analyses of inequalities related to cancer therapeutics/care and prevention have shown important disparities between and within countries, including those with high economic standards [1]. The conference explored overcoming these differences despite the relatively unequal financial situation across countries.

Over the last decades, the impressive development of basic biological and technological research has offered unexpected clinical/prevention research opportunities. Unfortunately, the translation to cancer therapeutics/care and prevention has been hindered by the sub‐optimal structural underpinning of clinical and prevention research. The latter appears particularly problematic for developing personalized/precision cancer medicine (PCM). This situation has been the subject of several investigations in Europe. Lately, a collective attempt to overcome some of these problems has materialized with the launching by the European Commission of the Mission on Cancer (MoC) [2] and Europe's Beating Cancer Plan (EBCP) [3]. Also, the USA Cancer Moonshot initiative is an illuminating example of attempting to bridge basic and clinical cancer research [4].

The conference addressed some important factors behind inequalities and how to improve equal access to cancer diagnostics, therapeutics/care and prevention. The European Union (EU) and its Member States' (MS') efforts are timely and of global interest. However, the success of these efforts will heavily depend on the strategies used to invigorate and interconnect the different modules of the cancer research continuum [5, 6, 7]. The conferences also addressed prevention and therapeutics/care including the specific issues low‐income countries face.

Basic and technological research sets the agenda for clinical and prevention translational cancer research innovation. A coherent bridging of basic and preclinical research and its integration with early clinical trials is a primary responsibility of countries with well‐developed basic research. Effective translational research covering clinical outcomes and health economics requires high‐quality, geographically dispersed infrastructures with good access to local patient populations [8]. These facilities can boost expertise and enable capacity building, especially in underprivileged areas. The establishment of suboptimal infrastructures for research will rather aggravate than mitigate inequalities.

There is a good reason for initiating the two comprehensive strategies, the USA Cancer Moonshot and the MoC in Europe. Despite advances in cancer research over decades, cancer incidence is still increasing, as well as its prevalence and the number of patients dying. Expanding possibilities for both prevention and therapeutics are expected to come from basic research; however, establishing a coherent and effective translational research continuum is a major challenge due to the complexity of exploiting the increasing number of promising innovations from basic research.

Following an introduction with the presentation of the main problems linked to inequalities, which are not only due to different economic differences between countries but also to the increasing complexity of cancer research, there were sessions on basic and preclinical research innovating the agenda for translational cancer research, examples of national structuring of cancer activities in EU member states and beyond, and on how to exploit innovation as the driver to reduce inequalities.

## 2 Welcome

**Fig. 1:**
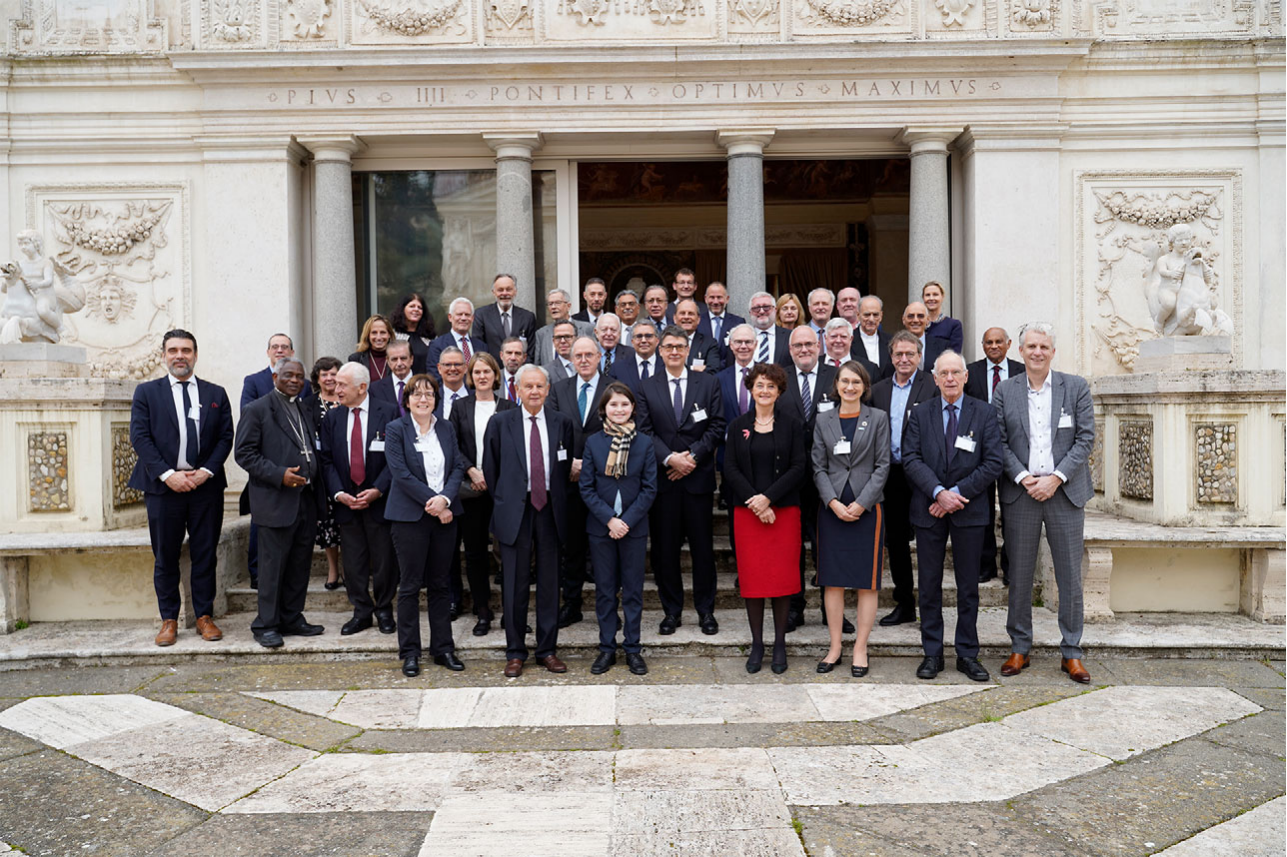
Photograph of participants of the conference by Gabriella C. Marino, PAS.

**Joachim von Braun**, President, PAS, and Cardinal **Peter Turkson**, Chancellor, PAS, welcomed the participants (Fig. 1), introduced the PAS and underlined the global problem of inequalities regarding healthcare and prevention. This view has also been expressed by His Holiness Pope Francis (Box 1).

Box 1 *‘Let us thank the Lord for the progress that medical science has made, especially in recent times; new technologies have made it possible to prepare therapies that are of great benefit to the sick; research continues to make a valuable contribution to eliminating old and new pathologies; rehabilitation medicine has greatly expanded its expertise and skills. None of this, however, must make us forget the uniqueness of each patient, his or her dignity and frailties. Patients are always more important than their diseases, and for this reason, no therapeutic approach can prescind from listening to the patient, his or her history, anxieties and fears. Even when healing is not possible, care can always be given. It is always possible to console, it is always possible to make people sense a closeness that is more interested in the person than in his or her pathology.’*

Message of His Holiness Pope Francis, 30th World Day of the Sick, February 11th, 2022.

In his welcome, **Michael Baumann**, President of the EACS, summarized the EACS' mission:

*Scientific knowledge*: by providing a forum for discussing the latest scientific trends in cancer research and reviewing opportunities and threats to further develop a collaborative cancer research environment in Europe.

*Policy*: By offering expert policy opinions on what is needed to diminish the burden of cancer and mitigate inequalities throughout Europe. It does this by organizing expert meetings and by publications accessible to a broad audience.

*Public Engagement and Dialog*: Interacting with other cancer organizations and charities to reach a consensus on relevant cancer issues and ‘speak with one voice’ to encourage coherent transnational initiatives to beat cancer.

*Excellence*: by promoting the quality and innovation of the cancer research continuum based on analyses of present needs for patients and risk individuals.

He finished by stressing that the EACS has actively advocated the launch of the MoC [5, 6, 7, 8, 9, 10, 11].

## 3 Opening session

**Julio Celis** from the EACS and DCI chaired the Opening Session which included five presentations by key stakeholders and an opening lecture on the main challenges for translational research aimed at PCM.

The European Commissioner for Innovation, Research, Culture, Education and Youth, **Mariya Gabriel**, opened the session by stating that around 2.7 million people in the EU are diagnosed with cancer yearly, with 1.3 million dying annually. Commissioner Gabriel stressed that while everyone should have the same right to high‐quality care, diagnosis and treatment, access to medicines, and hope of survival, today, inequalities in beating cancer still exist in the EU. Therefore, efforts must be intensified to address these disparities.

Commissioner Gabriel presented the Horizon Europe MoC, which offers a new way to address challenges in beating cancer by creating a portfolio of research and innovation‐coordinated actions combined with new forms of governance and collaboration. MoC works with European citizens, raising their awareness and encouraging their engagement, thus striving for co‐creative solutions that respond to the actual needs and benefits of all citizens at risk of cancer and cancer patients living with and beyond cancer. The Commissioner stressed that with EU initiatives on cancer, the European Commission (EC) aims to improve the lives of more than 3 million people by 2030 through prevention, better treatments and improving quality of life for patients and their families.

Commissioner Gabriel outlined key achievements of the MoC. First, she underlined that 30% of the world's stored data is produced by health systems, providing a unique source to advance understanding of cancer, yet, the health sector is lagging in exploiting that potential. Therefore, the MoC flagship European Federated Cancer Research Data Hub, UNCAN.eu (UNderstanding CANcer), will combine patient health data with research data at an unprecedented scale, using existing efforts worldwide. Second, the Commissioner mentioned the new EU Network of National Cancer Hubs, which will make it easier for MS to provide citizens with screenings and cancer care that meets the standards set by European guidelines and quality schemes. Through this EU Network, the aim is to ensure access to state‐of‐the‐art cancer diagnosis and treatment for 90% of the population in the EU by 2030.

Finally, the Commissioner flagged the real experiences of cancer patients, survivors, family members and caregivers behind the statistical figures. She spoke about her engagement with youth through the newly established European Network of Youth Cancer Survivors. The Network brings together over 40 partners from 25 European countries. It fosters social engagement, peer and mental health support and knowledge exchange to improve the quality of life and the care of children, adolescents and young adult cancer survivors.

Considering that Commissioner **Stella Kyriakides** having responsibility for the EBCP, could not attend the meeting, **Alberto Costa**, from her cabinet, provided a document addressing current developments of EBCP.

Through the EBCP, the EU aims to deal with all aspects of the disease pathway: prevention, early detection and treatment, and the quality of life of those affected by cancer. Since its launch in 2021, this first‐ever comprehensive EU‐wide Cancer Plan has delivered numerous actions and measures in all pillars of the Plan. For example, to improve standards of care in the EU, work has established an EU Network linking National CCCs running by 2025 for the first time.

In health‐related quality of life, an EU‐wide process was launched to address fairness in access to financial services for people with a history of cancer – the so‐called right to be forgotten. This action is the first step toward establishing the first‐ever EU Code of Conduct to ensure equal access to financial services for people with a history of cancer.

The latest of the EBCP milestones, and a watershed moment, was the agreement at the EU level of new, modern and science‐based recommendations for cancer screening – the first such update in 20 years. With more targeted and broadened routine screenings for an increasing number of cancer types, we will have a real chance to increase earlier detection across the EU and give health professionals the best possible tools to save lives and administer the best treatments.

At the beginning of the year, a new European Cancer Imaging Initiative was launched to use better the power of data and digital technologies, such as Artificial Intelligence (AI), to detect and address cancer, another crucial resource on which medical professionals and researchers can rely to be one step ahead of the disease.

And looking ahead, over 30 actions will be presented over the coming year. It will include work on updating the 4th edition of the European Code against Cancer (ECAC) based on the latest scientific evidence and recommendations to improve health literacy. A project is also ongoing to develop an EU Mobile App for Cancer Prevention, expected to be launched in 2024 and will help promote the ECAC among citizens.

Moreover, the EC will propose a Prevention Package later this year, including a proposal to update the 2009 Council Recommendation on smoke‐free environments and contribute to creating a Tobacco Free Generation in Europe by 2040. At the same time, the EC will also propose a Council Recommendation on vaccine‐preventable cancers to help increase the vaccination uptake against Human Papilloma Virus (HPV) and the Hepatitis B virus.

The EBCP has an unprecedented budget of €4 billion to finance these activities. Under the EU4Health program, more than €400 million worth of EU funding has been programed until now for cancer. It includes over 100 million for the launch so far of 28 ambitious projects touching on all areas of the EBCP—from prevention to early detection, from treatment to quality of life after cancer.

Finally, global cooperation is also key to tackling cancer. The EC has so far engaged in a discussion on collaboration with the USA Cancer Moonshot program and is now setting up an EU‐US Health Taskforce. These discussions will impact not only patients and families in the EU and US but also worldwide, and we are interested in hearing from experiences from other countries with important actions on cancer.

These are only some of the developments that will help to mobilize EU efforts to prevent cancer, improve equal access, and improve the lives of all those touched by this disease.

Next, Elisabete Weiderpass*, Director of the International Agency for Research on Cancer (IARC/WHO), Lyon, France, spoke about inequalities in cancer care and prevention.

She emphasized that over this century, cancer will become the leading cause of premature death worldwide and the single most important barrier to further gains in life expectancy. According to IARC's estimates, in 2020, there were 19.3 million new cancer cases and almost 10 million cancer deaths worldwide [12]. Cancer does not affect the world population uniformly. Compared to the US and Europe, the share of new cancer cases and cancer deaths is higher in Asia and Africa because of the different distribution of cancer types and more elevated case fatality rates in these regions. Globally, new cancer cases are expected to increase from 19.3 million in 2020 to an estimated 30.2 million in 2040. The greatest increases are predicted in lower‐resource countries currently assigned a low Human Development Index **(**HDI), a composite indicator of life expectancy, education, and gross domestic product per person. The above highlights the clear reality of increasing inequalities between countries. Efforts to plan, implement and evaluate prevention programs, particularly HPV vaccination, tobacco control, and dietary and lifestyle recommendations, must be considered greater priorities in low‐ and middle‐income countries. Access to appropriate, affordable, and equitable treatment will also be crucial in low‐resource countries. In Europe, recent IARC studies revealed that between‐ and within‐country cancer inequalities were associated with education levels and reflected inequalities in the availability, access and uptake of effective preventive programs, such as screening [13]. These findings call for systematic measurement, monitoring and action on the substantial socioeconomic inequalities in cancer in Europe.

*Disclaimer.

Where authors are identified as personnel of the International Agency for Research on Cancer/World Health Organization, the authors alone are responsible for the views expressed in this article; these views do not necessarily represent the decisions, policy or views of the International Agency for Research on Cancer/World Health Organization.

**Douglas Lowy**, Deputy Director of the National Cancer Institute, Bethesda, Maryland, USA, next addressed health disparities with the USA Cancer Moonshot and beyond. The presentation began by noting that the USA Cancer Moonshot and the ECBP share two critical goals: (a) improving the options for preventing, screening, detecting, and treating cancer through research and (b) narrowing and eliminating the disparities in the unequal cancer incidence and outcomes within the populations of EU countries and the USA that are mainly attributable to unequal access to cancer care and control.

Beyond this EU‐centric and USA‐centric framework, cancer disparities between high‐income countries (HICs), such as the EU and USA, and low‐ and middle‐income countries (LMICs) are generally much greater than those within HICs. Furthermore, it is predicted that most of the increase in cancer incidence and mortality over the next 20 years will occur in LMICs. It is, therefore, important to consider approaches that can reduce the global disparities in cancer and those within HICs.

Although several population‐wide interventions can be envisioned, two were highlighted. The first is to even more strongly promote efforts to reduce tobacco consumption worldwide, as this carcinogen is responsible for around one‐third of all cancer and is a major contributor to serious cardiovascular and pulmonary disease. The second is to reduce the risk of cervical cancer, one of the most common female cancers in LMICs, through HPV vaccination* and cervical cancer screening.

The overall potential for technology to help reduce cancer disparities was also noted. For this to happen, it is important from the outset to consider the impact of new technology on cancer disparities.

*Disclosure: The author is a developer of the technology that underlies the HPV vaccine.

**Francesco de Lorenzo**, president of the European Cancer Patient Coalition (ECPC), Brussels, Belgium, presented the next talk covering patient perspectives. Various unacceptable inequalities exist across Europe regarding cancer therapeutics, care and prevention, between and within countries, including those with high economic resources. Such inequalities include:

- Access to curative cancer treatments.
- Extreme variability and accessibility to cancer screening services.
- Fragmented or missing cancer rehabilitation services.
- Poor governance.
- Major organizational, structural, and fiscal deficits in health systems operation.
- Lack of cancer survivorship plan and care.
- Lack of patient‐accessible, accurate and up‐to‐date cancer and health care information.

In addition, we have to look further than healthcare and recognize that the existence of socioeconomic inequities is an indication of the existence of cancer inequities,

The vision of the ECPC, acting as the voice of cancer patients, is to work toward a Europe of equality, where all European cancer patients have timely and affordable access to the best treatment and care available throughout their life.

ECPC has been raising awareness of the key issues that lead to disparities in cancer care for many years. ECPC has worked tirelessly for the implementation of National Cancer Control Plans in Europe, aiming to reduce the burden of cancer. Such National Cancer Control Plans can be instrumental in reducing the cancer burden in Europe through their harmonization with the provisions of the novel strategy of EBCP. It marks a new era in cancer care and prevention, ensuring access to essential medicines and innovation to be reached through the Pharmaceutical Strategy for Europe, including the revision of the legislation to provide access to affordable drugs for patients and addressing unmet medical needs, as well as the timely adoption for the Health Technical Assessment (HTA) Regulation. Member States now can benefit from the Recovery and Resilience Fund (RRF) and the EU Structural and Cohesion Funds to reduce inequalities in access to health services and to offer to all their citizens appropriate screening, care, follow‐up and quality of life to move from ‘how long people live after diagnosis’ to ‘how well people live with a diagnosis’.

Finally, the Opening lecture on the Main challenges for translational research aimed at personalized/precision cancer medicine was delivered by **Ulrik Ringborg** from the Cancer Center Karolinska, Stockholm, Sweden. Inequalities in research contribute to inequalities in cancer therapeutics/care and prevention, and today there are pronounced disparities between poor and wealthy countries and also within wealthy countries. Basic biological and technological research discoveries demand increased infrastructure support for clinical and prevention research activities. The EU EUROCAN + Plus project (2005–2007) defined translational research as a coherent cancer research continuum from basic/preclinical to clinical research and further to implementation in healthcare for outcomes and health economics research. Initially, translational research for cancer therapeutics/care aimed at bridging the gap between basic and clinical research, and later the gap between clinical research and implementation became visible.

**Fig. 2:**
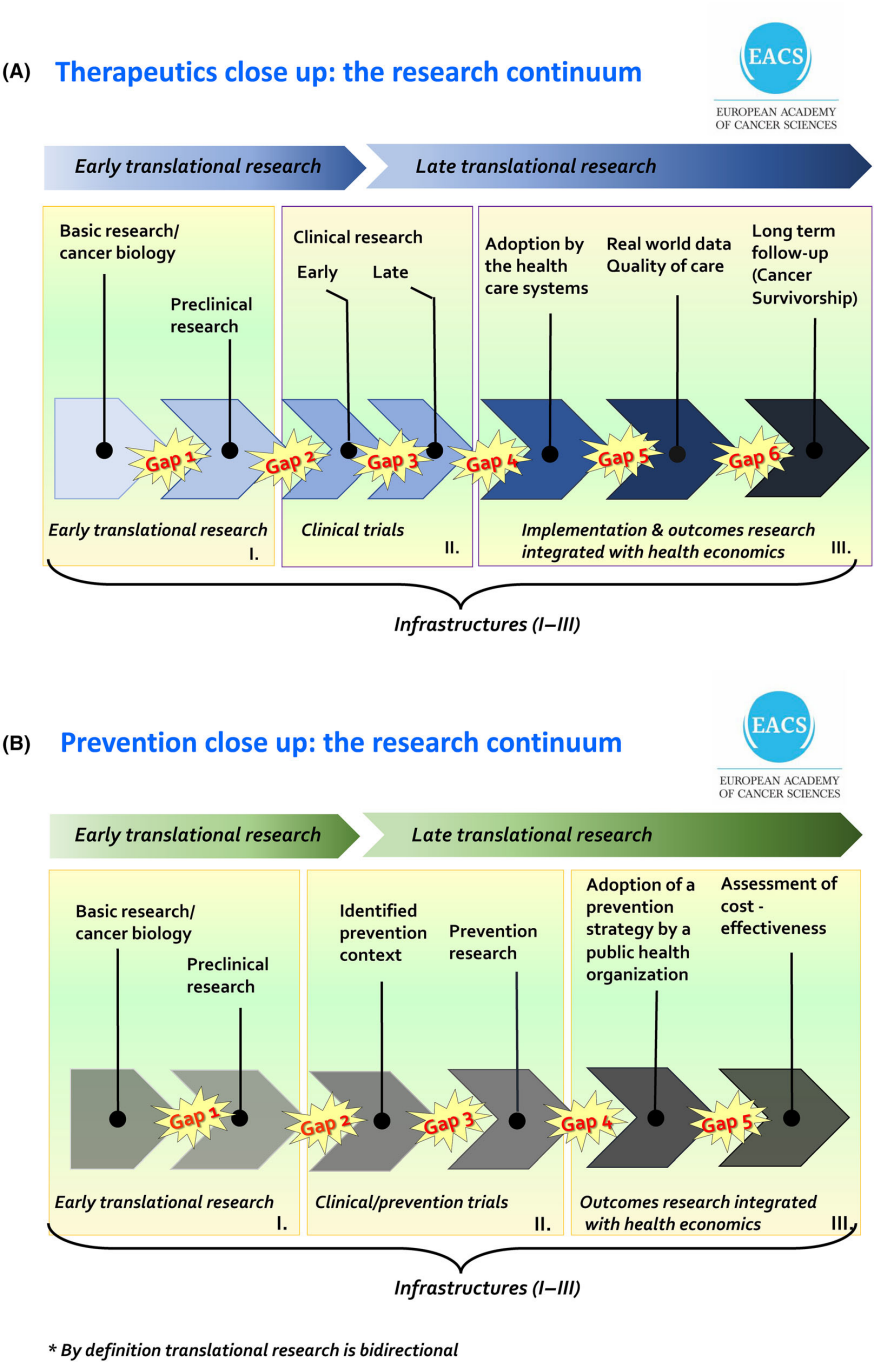
The Cancer Research Continuum for therapeutics/care and prevention. An outline of the relevant modalities and gaps within the cancer research continuum for (A) creating cancer therapeutics and (B) for cancer prevention.

Over time more gaps have been identified (Fig. 2A) and bridging the gaps for the coherence of the research continuum is an increasing challenge. A similar research continuum for cancer prevention should cover primary and secondary prevention (Fig. 2B). Translational research is needed to innovate all cancer therapeutics/care and prevention components. The accelerating rate of discoveries in basic biological and technological research makes the preclinical research aiming at new proofs of concept for early clinical trials and prevention contexts still more important.

Basic and preclinical cancer research sets the agenda for translational cancer research. Basic biological and technological research discoveries are expanding rapidly, while clinical and prevention cancer research is time‐consuming and complex. Preclinical research supports the development of proof‐of‐concept clinical trials and prevention contexts for intervention research. Reverse translational research increases the need for basic and preclinical research. More close interactions between basic, preclinical and clinical researchers are needed for prioritizations and expansion of preclinical research is urgently needed to prepare for early clinical and prevention research.

For a coherent clinical cancer research continuum, a critical mass of infrastructures and patients is required for next‐generation clinical trials on molecularly stratified patients. With potential interest for implementation in the healthcare practice changing/treatment optimization, randomized comparative trials to assess clinical efficacy and health economics are the next steps before implementation research to assess clinical effectiveness and health economics. Health‐related quality‐of‐life should be an integral part of the cancer research continuum, also after long‐term follow‐up.

Integration of cancer therapeutics/care and research in CCCs is mandatory for developing translational cancer research. The responsibility of a CCC for a defined geographic area is critical to offer more patients high‐quality care and access to clinical research. At present, there are 47 accredited CCCs in the EU, but about 100 (each with responsibility for maximum 4–5 million inhabitants) are needed to decrease present inequalities.

The drug development process is incomplete when the final steps (practice‐changing trials/treatment optimization studies followed by implementation research) are missing, with insufficient information on clinical effectiveness and health economics (survival benefit, health‐related quality of life evaluation, cost‐effectiveness) as a consequence before recommended implementation in the healthcare. Anticancer agents with insufficient antitumour effects increase costs without expected positive impact. Potential side effects may increase costs further for healthcare, which is also an ethical problem from the view of patients.

There is an ongoing process to develop a molecular classification of tumors and stratification of patients for agnostic clinical trials. Most patients are diagnosed and treated outside the CCCs, while infrastructure support for the stratification of patients (molecular pathology, omics technologies) are available at well‐developed cancer research centers. Sharing patients´ data/biological samples and infrastructures at the national and international levels is necessary for achieving the critical mass to develop PCM. Without sustainable collaborative networks, suboptimal innovation, and inequalities within and between countries will increase. The latter has also a bearing on the health‐related quality of life research including supportive care, rehabilitation, psychosocial oncology and palliative care.

In summary, a coherent cancer research continuum will increase innovation toward cost‐effective therapeutics/care and prevention and offer relevant tools for prioritization. Sharing resources to develop research on molecularly classified tumors (centralized infrastructures for molecular pathology and omics technologies, treatment of patients where they live) will make advanced clinical research available for more patients and mitigate inequalities.

### 3.1 Discussion and conclusions

In general, questions dealt with (a) the complexity of the MoC set‐up and how the EACS could help make the MoC and the EBCP as effective as possible, (b) the lack of funding instruments for translational clinical and prevention research, (c) how best to coordinate efforts in a pan‐European manner and strengthen the EU association with other countries at the frontier of science like the USA. Research is a competence of the EU, but health is not; how best to align priorities and policies?

Other questions included what measures known to work could be quickly taken to reduce cancer incidence, how we can increase the funding allocated to prevention, the role of patients and civil society in escalating international cooperation, and how can the EACS and the cancer community help create the mission hubs which will be established in all MS.

## 4 Session 1: Innovative basic, preclinical, and technological research – The agenda for translational cancer research

### 4.1 Chair: Edith Heard, European Molecular Biology Laboratory (EMBL), Heidelberg, Germany. Conclusions were summarized by Jan Korbel and Katherine Silkaitis, EMBL's Strategy Team.

**Olivier Michielin**, CHUV Centre Hospitalier Universitaire Vaudois, Lausanne, Switzerland, opened the session by illustrating how **Current cancer biology and immunology drive the development of preclinical and early clinical research**.

In the last decades, several technological breakthroughs have profoundly transformed our capacity to interrogate the complex biology of cancer. We now have a much deeper understanding of the principles that govern tumor growth and resistance to therapy and, in particular, the role of the tumor micro‐environment in sustaining tumor cells. Such research has led to many new targets within the tumor cells or the tumor microenvironment. Targeted therapies with small molecule inhibitors that block oncogenic pathways or immuno‐therapies that promote effective anti‐tumor immunity are two examples of direct translation from basic biology research to innovative clinical opportunities for our patients. Despite this remarkable progress, most patients will still recur, and overall survival remains poor in many metastatic settings. One possible approach to improve patient benefit is to tailor treatment selection to the complex biology of the tumor within precision oncology or precision immuno‐oncology programs. The rich translational data from tumor biopsies, including genomics, transcriptomics, or proteomics, contain information that can help guide treatment decisions after standard of care approaches fail. Large data sets and advanced data science approaches are needed to efficiently pilot such precision oncology programs that flourish worldwide, providing new therapeutic options for our patients.

Inequalities can be present at every critical step in this process. Indeed, immunological research can focus on HLA types that are more prevalent in industrialized countries. Artificial Intelligence training data sets can similarly have an over representation of university hospital patients compared to the general population and, therefore, perform better for these patients. The scientific and medical community must be aware of these risks to minimize these potential pitfalls and their resulting inequalities.

**Olli Kallioniemi**, Science for Life Laboratory and the SciLifeLab‐KAW Data‐Driven Life Science Program, Department of Oncology & Pathology, Karolinska Institutet, Stockholm, Sweden, expanded on the theme of data science and precision oncology: **A Data‐Driven Approach to Transform Millions of Multi‐Omics Data Points into Actionable Precision Cancer Medicine: An Example of How Artificial Intelligence (AI) Can Promote Equality in Cancer Diagnostics and Care**.

Disparities in cancer research and care exacerbate health inequalities across the globe. As part of a national program on data‐driven life science, we aim to promote the transformative potential of AI and data‐driven life science in advancing translational cancer research and developing novel molecular oncology diagnostics.

AI techniques, like machine learning and deep learning, enable large‐scale data analysis, pattern recognition, and predictive modeling. These techniques can be applied to gain novel insights into cancer biology, diagnostics, and treatment. Integrating multi‐omics data, including genomics, transcriptomics, proteomics, metabolomics, and functional drug testing using AI tools, can facilitate the development of personalized cancer therapies and improve patient outcomes. However, the high cost and complexity of multi‐omics data pose significant practical challenges in translating these findings to practical clinical applications, particularly in resource‐limited settings.

The Weinberg–Hanahan cancer hallmark concept defines a limited set of independent biological properties that play a role in cancer development and progression. We have taken the spirit of the cancer hallmark paradigm to condense complex multi‐omics properties of cancer into a limited number of unbiased, quantifiable properties that we call data‐driven hallmarks (DDHMs). Our study is based on acute myeloid leukemia (AML) samples profiled with multi‐omics and tested for the efficacy of 500+ drugs in high‐throughput *ex‐vivo* (T. Erkers *et al*., manuscript in preparation). Altogether, almost 100 million data points were acquired and condensed into about a dozen dimensions of variability, which we call data‐driven cancer hallmarks (DDHM).

Each cancer patient is characterized by a unique combination of independent and potentially druggable DDHMs. This contrasts with traditional cancer stratification, where each patient is assigned to one specific subgroup defined by, for example, oncogene drivers. Most DDHMs are driven by data types other than genomics, emphasizing the importance of incorporating multi‐omics data into cancer research and diagnostics. DDHMs can predict high‐risk AML as well as specific drug response vulnerabilities. Therefore, applying the DDHM concept provides an opportunity for precision medicine diagnostics and individualized therapies.

In a broader cancer research and diagnostics context, by incorporating AI technologies and data‐driven approaches, we can develop targeted diagnostic panels that are affordable and fit the clinical *in vitro* diagnostics requirements but provide similar power and information as comprehensive and expensive multi‐omics profiling studies. Our proof of concept thus bridges the gap between costly data‐intensive academic research studies and clinical precision medicine needs for defining individually tailored drugs and drug combinations for cancer patients. This approach could eventually foster more inclusive and accessible global healthcare, paving the way for innovative cancer treatments and improved patient outcomes.

In the subsequent talk, **Edward M. De Robertis**, Ayse Y. Azbazdar and Nydia Tejeda Muñoz, from the David Geffen School of Medicine at the University of California, Los Angeles, USA, focused on **Cell Biological Approaches in Cancer Treatment**.

The peculiar cell biology of cancer cells can potentially be used for non‐targeted cancer therapies. One of the main pathways activated in cancer is canonical Wnt signaling. Upon binding to their plasma membrane receptors, Wnt‐secreted proteins inactivate a destruction complex that normally degrades the key regulator β‐catenin. Upon stabilization, β‐catenin translocates into the nucleus and activates the transcription of proliferation‐inducing target genes. In cancer, constitutive Wnt signaling can be achieved, for example, by loss‐of‐function mutations in the components of the degradation complex adenomatous polyposis coli (APC) and Axin1, which are major tumor suppressors in colorectal and hepatocellular cancer, respectively.

Work from our laboratory has shown that in the presence of Wnt, receptor complexes are endocytosed and translocated to the intraluminal vesicles of multivesicular bodies (MVBs). The receptors are bound to the components of the destruction complex, particularly the key enzyme Glycogen Synthase Kinase 3 (GSK3). The sequestration of GSK3 inside membrane‐bound organelles stabilizes the degradation of many phosphodegrons in cellular proteins (called Wnt‐STOP), the most prominent of which is β‐catenin. The formation of MVBs is part of the normal membrane trafficking machinery: all plasma membrane proteins must transit through MVBs to reach the lysosomes, where they are degraded. Thus, the Wnt signaling pathway co‐opted normal cellular trafficking.

When HeLa cells are treated with Wnt3a protein for short periods (5–20 min), large vesicular structures corresponding to GSK3‐containing MVBs are formed in lamellipodia, particularly at sites of focal adhesions. Adding Bovine Serum Albumin‐dequenched (BSA‐DQ) reveals that Wnt rapidly stimulates protein uptake and subsequent targeting to lysosomes, where the extracellular protein is quickly degraded. The BSA‐DQ reagent specifically fluoresces after the BSA protein is degraded in lysosomes. The increase in endocytosis results in important cellular metabolic changes, including increased acidification of lysosomes using Lysosensor probes.

The increased dependence on extracellular protein in Wnt‐stimulated cells is due to elevated macropinocytosis (Gr., *pinein*, to drink), an actin‐mediated cell‐drinking type of endocytosis that involves the engulfment of large fluid vesicles several microns in diameter. Macropinocytosis requires the activity of the Na/H^+^ exchanger in the plasma membrane. After Wnt treatment, Axin1 mutation, or APC mutation, massive macropinocytosis can be seen in the apical regions of the plasma membrane. This represents a large effort for the cell and a point of vulnerability. The colorectal cancer (CRC) cell line SW480 has high constitutive nuclear β‐catenin activity due to APC mutation. Interestingly, β‐catenin signaling can be inhibited by blocking macropinocytosis with the Na/H^+^ exchanger inhibitor EIPA or the human diuretic Amiloride within only 2 h of treatment.

Tumor promoters are substances that do not cause cancer by themselves nor mutate the DNA, but following exposure to an initiator mutagen, promote cancer development. It was known that the tumor promoter phorbol ester PMA/TPA increases macropinocytosis. Data will be presented showing the effect of increasing macropinocytosis by treating cells with tumor promoters. Results from CRC human arrays immunostainings indicate that as malignancy progresses, β‐catenin increases, the lysosome marker Vacuolar‐ATPase (V‐ATPase) is markedly increased, and GSK3 levels are decreased. The sequestration of GSK3 and lysosomal activation also helps explain the genesis of the initial Wnt signal that triggers axial development in the *Xenopus* embryo.

These basic studies imply that strategies potentially interfering with Wnt‐induced macropinocytosis, multivesicular body formation, or lysosome activity may decrease cancer progression [14, 15, 16, 17]. For example, Na/H^+^ exchanger inhibition with Amiloride, lysosomal activity by V‐ATPase inhibitors or lysosomotropic agents, or macromolecular conjugates (e.g., Abraxane) targeting macropinocytosis could in principle provide non‐targeted therapies for a wide spectrum of Wnt‐driving cancer mutations – such as APC, Axin, RNF43, ZNRF‐3, R‐Spondin 2 or 3 translocations, and activating β‐catenin point mutations. However, so far, the targeted therapeutics have been successful, as we will hear in the next presentation.

**Charles L Sawyers**, Howard Hughes Medical Institute and Memorial Sloan Kettering Cancer Center, New York, USA, discussed **Molecularly Targeted Therapies: from the Laboratory to the Bedside** and emphasized the value of molecularly targeted therapies in cancer patient care.

Decades of basic science and extensive tumor sequencing efforts have firmly established that cancer is a disease of mutations. Initial hints that these mutations might be targeted directly with new classes of drugs date back to estrogen receptor therapy in breast cancer and retinoic acid receptor therapy in acute promyelocytic leukemia, but the concept was crystalized by the spectacular success of ABL kinase inhibitors in chronic myeloid leukemia and EGFR inhibitors in lung cancer. These examples sparked the Cancer Genome Atlas (TCGA) project in the US and the International Cancer Genome Consortium (ICGC) effort to sequence thousands of tumors ‐ a hugely successful BIG science investment that yielded a plethora of new data on cancer driver mutations. The discovery of these new drivers sparked huge investments in cancer biology research in academia and drug development by biopharma, with ~100 new molecularly targeted cancer drugs approved over the past 20 years.

Consequently, the cancer landscape today is viewed as hundreds of smaller diseases, with the understanding that complex molecular testing is now required to obtain an accurate diagnosis to ensure the right drug for the right patient. This landscape change, while welcome, requires changes in the infrastructure we use to conduct clinical trials by incorporating molecular data to assess trial eligibility through pre‐screening large numbers of patients in advance. Cancer centers (and consortia of cancer centers) provide a logical path forward through real‐time sharing of genomic and clinical outcome data. Sharing of patient data (genomic and clinical) at the necessary scale is challenging, but initiatives such as Project GENIE, based in the US under the leadership of the American Association for Cancer Research (AACR), demonstrate the feasibility of generating these critical databases. However, we need to be cognizant about the generalizability of insights gained from these cancer center consortia, which currently are populated largely by patients of European ancestry. Going forward, it is critical that we increase the representation of non‐European ethnicities through proactive inclusivity efforts.

**Mechthild Krause**, from EACS and Carl Gustav Carus University Hospital, Dresden, Germany, spoke on the **Technological development for innovation in radiation oncology**.

Today, in Western Europe, every second cancer patient can be cured of his/her disease using modern treatment options. Radiotherapy plays a major role in this regard and is involved as sole treatment or as part of a multidisciplinary treatment in about half of all cancer cures. If treatment is intended to relieve symptoms, most patients require radiotherapy.

Over the last decades, radiotherapy has made substantial progress in the technique of radiotherapy, e.g., the treatment of irregular volumes, image guidance or stereotactic radiotherapy, where high doses are applied to usually very small targets. Biological imaging has been introduced in radiotherapy, and the combination of radiotherapy with drug treatments has developed in the 1990s and has been further optimized over the years. Specific beam qualities like protons are increasingly evaluated for their advantage in patient outcomes.

With novel technologies for the characterization of tumor microenvironment and molecular and genetic parameters, individualized treatment schedules are developed, where the use or the characteristics of radiotherapy schedules will be adapted to individual tumor features. While today, we treat large groups of patients in the same way and make treatment decisions solely on tumor histology, stage, and a few patient‐related factors; an increasing patient stratification is expected in the future, resulting in smaller and smaller patient groups with identical treatments. An example is the HPV infection in head and neck squamous cell carcinoma, which has resulted in better radiotherapy outcomes [18]. It is currently used in radio oncology trials to identify patient groups that could profit from de‐escalating the treatment. Such biomarkers can also be specified with imaging, e.g., the correlation of tumor hypoxia detected with positron emission tomography (PET) with worse outcomes of radiotherapy [19, 20]; this parameter is evaluated in ongoing interventional trials.

Examples of technology development are proton radiotherapy or integrating magnet resonance tomography into linear accelerators (MR‐Linac). Proton radiotherapy can potentially protect healthy normal tissues behind the treated volume and is currently used in some accepted indications like pediatric cancers or base of skull cancers in a few specialized centers worldwide. MR‐Linacs may have advantages in treating moving targets, as treatment plans can be adapted during a radiotherapy fraction. Identifying patient groups with improved outcomes after such treatments is a matter of ongoing trials.

A major obstacle to radiotherapy is the low access to (standard) radiotherapy facilities in low and median‐income countries [21], preventing large parts of the population from standard medical care. At the same time, some very frequent cancers with high incidences in these countries are under‐researched, and treatment has thus not been improved at the same speed as for high‐incidence cancers in high‐income countries.

In conclusion, radiotherapy will increasingly develop into an individualized treatment, including biological stratification of patients by tumor features. This requires the availability of molecular techniques, modern imaging and genetics and will, in the future, lead to AI‐based treatment decisions. Technological advances, e.g., in proton radiotherapy or MR‐integrated linear accelerators, are, with few exceptions, still within clinical trials to generate evidence, but will lead to AI‐based treatment adaptation and potentially the use of robotics in the midterm future. Low‐and middle‐income countries need adequate access to standard radiotherapy techniques to reach treatment outcomes comparable to high‐income countries and to improve treatment of cancers that are of high incidence in their region. The low support of these countries in radiotherapy access and clinical trials will otherwise lead to a further loss of connection to the next steps of radiotherapy improvement (e.g., personalized treatment, AI methods etc.).

**Jan Korbel**, from EACS and the European Molecular Biology Laboratory (EMBL), Heidelberg, Germany, discussed **The role(s) of data science for future biological and clinical cancer research** and emphasized opportunities for AI‐guided cancer pathology.

Modern machine learning, frequently referred to as simply AI is likely to revolutionize cancer diagnosis based on image analysis in the future. Pathology based on H&E images is currently the gold standard for cancer diagnosis. Still, it can be challenging for pathologists, especially in regions of the world that need access to well‐trained pathologists. AI‐based image analysis can help address these challenges and could be ‘paired’ with, and ultimately guide, classifications made by trained clinician experts. This could improve the accuracy of cancer diagnoses and help reduce the burden on pathologists – including in low‐resource settings, which could help reduce healthcare disparities in the future.

Modern machine learning also has the potential to improve decision‐making in cancer patient management by integrating heterogeneous data from different sources. Cancer patient management increasingly relies on integrating data from multiple sources, from electronic health records to molecular data types. This data heterogeneity presents a key challenge for clinicians who must make decisions based on the available data, highlighting another setting where AI algorithms could help overcome challenges in translational research and clinical care.

Dr Korbel cautioned that the challenges and risks of applying advanced machine learning techniques in cancer patient management should not be underestimated. For example, AI models developed in high‐income countries may not translate well to, and thus be ineffective for, patients in the Global South, where there are differences in genetic, environmental, and socioeconomic factors affecting cancer incidence. In the future, it will be critical to address these biases by developing AI models that are trained on and represent more diverse populations. This could be achieved by enhancing large‐scale data sharing mechanisms and applying sophisticated AI methodologies to globally generated datasets. Another concern is the ‘black box’ nature of AI models, which do not disclose the reasoning behind their ‘decisions’, resulting in a lack of transparency that could pose challenges for clinicians to comprehend the underpinnings of AI‐driven medical decisions. To address this, ongoing research is focused on creating more ‘explainable AI’ models, which provide a more interpretable and transparent decision‐making process, aiming to advance translational research and improve cancer patient care.

Finally, to conclude the session, **Edith Heard** of the PAS and the European Molecular Biology Laboratory (EMBL), Heidelberg, Germany, discussed how **Basic research innovations advance preclinical and early clinical research**.

EMBL research enables a deeper understanding of cancer progression and contributes to better diagnosis and treatment. Its researchers are driving forward AI‐based and computational methods to integrate and extract biological insights from multi‐modal omics datasets. Dr Korbel, for example, highlighted the potential of AI‐guided cancer pathology to transform cancer diagnosis through image analysis. In her presentation, Prof Heard highlighted computational analysis from EMBL that laid the ground for early detection of tumors through blood samples. This work by EMBL complements the important research by Drs Michielin and Kallioniemi, among many others. Together, advances incorporating AI‐driven methods and analyses can expand the data used in diagnosis and treatment. This ability to study cancer at scale – scale in types of data and quantity of data – can democratize personalized oncology.

Beyond AI, EMBL researchers are developing new model systems to study cancer development and treatment. As Prof. Heard discussed, these include *in vitro* engineered tissues and organoids used to investigate topics ranging from tumor drug transport to stem cell differentiation. These can further contribute to precision oncology, opening a future that allows everyone the opportunity to have a cancer diagnosis and treatment personalized to their unique disease.

The research carried out at EMBL is part of its current 5‐year scientific program, ‘Molecules to Ecosystems’, which aims to advance our understanding of human health, considering that human life happens in the context of numerous ecosystems. For example, the human microbiome is a key component of the program, and EMBL contributes to our understanding of its relationship to cancer. To illustrate the importance of the microbiome in cancer, Prof Heard discussed work that identified a fecal microbiota signature that could be used in non‐invasive pancreatic cancer screening. She also described ongoing research into the interactions between colon cancer, immune cells, and microbiota that can provide insight into differential responses to immunotherapy treatment.

Another key component of EMBL's scientific program, and a component of all of EMBL's research, is ensuring that data and research results are freely accessible. EMBL's commitment to open data can be seen in many initiatives, such as a public‐private partnership that makes drug‐target associations publicly available and through policies that ensure all research is published as open access with accessible datasets.

Prof Heard emphasized that open data and research are critical factors in reducing inequalities in cancer research, diagnosis, and treatment. The downstream effects of sharing data and research findings are innumerable and robustly demonstrated by speakers in this session. Precision oncology, as presented by Dr Michielin, the data‐driven hallmarks of Dr Kallioniemi, and mutation insights gained from cancer center consortia, as discussed by Dr Sawyers, to name only a few, are underpinned by data sharing and/or publicly accessible data.

### 4.2 Discussion and conclusions

Many speakers at this session also highlighted the challenges of advancing cancer research and care when much of the patient populations and data are from European or non‐globally representative populations. This awareness is critical to ensure that the inequalities in medicine do not continue to propagate and leave vulnerable populations behind. While there is much that needs to be done to reduce disparities in cancer research and treatment – which ranges from increasing patient sampling from non‐Western populations to ensuring access to standard technologies – synergistic improvements to global human health, even beyond cancer, can be realized by ensuring that such data and research outputs are globally and freely accessible.

Other critical questions. How to develop mechanisms to prioritize innovations for early prevention and clinical research is a key issue. Interaction between basic and clinical researchers is an unmet need. The CCC has an important function since it covers the entire cancer research continuum and should be open for collaborations with basic cancer research centers. Educational activities should be expanded by exposing basic researchers to the most important clinical questions, and clinical researchers to basic and technological research development. Preclinical research has an important role and needs more focus. Well‐prepared proof of concept initiatives for prevention and therapeutics will make early clinical trials and further translational research more effective.

## 5 Session 2: Current situation and examples of national structuring of cancer activities in EU member states and beyond

### 5.1 Chair: Alexander Eggermont, University Medical Center Utrecht & Princess Máxima Center for Pediatric Oncology, Utrecht, The Netherlands

**The concept of Comprehensive Cancer Center – multidisciplinary cancer care and innovation** was presented by **Simon Oberst**, Organisation of European Cancer Institutes (OECI), Brussels, Belgium.

There is now increasing evidence in the literature of better patient outcomes for patients treated in CCCs (especially in the US [22, 23]), although establishing precise cause and effect is clearly very complex. The OECI, through having 65 of the largest cancer research centers in its accreditation program (aside from those in Germany who have their own accreditation system) is finding that while clinical multidisciplinarity is well embedded, governance structures are often not optimal to guarantee the best integration of research and care [24]. As a neutral finding, based on numbers and impact of peer‐reviewed publications and numbers of clinical trials open, cancer research is concentrated in around 50 CCCs in Europe.

Several baskets of issues are concerning to leaders of CCCs in Europe: workforce issues (recruitment and retention; burnout; vacancies; skill‐mix; continuous professional education); implementing PCM (molecular tumor boards, data integrity; bioinformatics; AI; precision biobanking); and developing new areas of research (e.g., prevention research; outcomes research; implementation science).

There are some outstanding examples of integrating research, education and care in OECI‐accredited CCCs. One such is the Cancer Research UK Cambridge Centre [25]. There the Programme Structure is a dynamic model which incentivises the involvement of more than 1000 clinical leaders, principal investigators and others to collaborate in either disease‐specific or discipline‐specific programs. Success of programs is rewarded with further funding and the opportunity to form ‘virtual institutes’ and finally physical institutes. Having high‐caliber program managers who arrange multiform meetings, colloquia, and projects between many professional groups are key to success.

The EU Joint Action of MS (CraNE) [26] aims to prepare for the creation of an EU network of CCCs. This would embrace the existing certified CCCs in OECI and in Germany. But there are challenges around the timing of implementation; the variety of sizes and models of cancer centers in EU states (or their existence at all), on the sustainable funding and overall purpose of the EU network, and the feasibility of reaching 90% of eligible patients by 2030.

The Lancet Oncology European Groundshot Commission on Cancer Research 2022 [27], based in part on comprehensive bibliometrics 2009–2020, showed that the EU MS who were members of the EU before 2004 (the ‘EU15’) have doubled cancer research outputs in those 11 years. However, those countries joining the EU after 2004 (the ‘EU13’) have shown slower‐growing outputs. The challenge therefore is how to build research capacity in central and eastern European member states through twinning and teaming efforts. Overall, per head of population, the study shows that Europe spends just over one tenth of the cancer research expenditure per head compared to USA. This shortfall must be addressed at national and EU levels. Furthermore, investment in cancer research undertaken in the EU is heavily weighted toward biology and innovation in diagnosis and treatment, which, while not surprising, dwarfs investment in prevention and survivorship (approximately 11% of the whole). This argues not for less investment in biology and treatment, but on the contrary, for more emphasis on outcomes research, prevention research, and implementation science.

**Development of a national research landscape in Germany. Michael Baumann**, Scientific Director and CEO of the German Cancer Research Center (DKFZ) and President of EACS, presented the German model as a successful example of structuring national cancer activities.

Germany has a highly segmented cancer care system including private practices, community hospitals, private clinics, and 36 university medical centers (UMCs). Cancer research is performed at medical faculties and non‐university research institutions. In 2003, the first multidisciplinary CCCs according to international standards were launched. Through a competitive, peer‐reviewed funding program of the German Cancer Aid, the network has grown to currently 15 accredited CCCs.

As a national laboratory in the Helmholtz Association, the DKFZ is Germany's largest biomedical research institution. Its more than 1300 scientists aim to reduce new cancer cases and cancer deaths through transformative research, educating the next generation of leaders, and translating results into the healthcare system, society, and innovative products. Because the DKFZ was founded in 1964 as a research institution without a cancer hospital, it has established strategic institutional partnerships with leading UMCs in Germany for translational and clinical cancer research. These partnerships are formally DKFZ branch offices that receive long‐term institutional funding for cancer research by the Federal Ministry of Education and Research (BMBF) and the federal states. Governance of the local preclinical (DKTK) and clinical (NCT) translational research sites is jointly performed by DKFZ and the local UMCs; national governance is provided by joint steering committees bringing together DKFZ, all partner sites and patient representatives.

To bridge the gap between basic cancer research and clinical cancer research, the BMBF and the respective federal states support the German Cancer Consortium (DKTK), which links the DKFZ with 11 UMCs and more than 20 academic and clinical research institutes at seven partner sites throughout Germany. The mission of DKTK is to conduct cutting‐edge bi‐directional (forward and reverse) translational cancer research. Since its inception in 2012, DKTK has successfully pushed frontiers to transform the cancer research landscape in Germany from a competitive toward a cooperative approach and to accelerate the transfer of basic scientific findings into clinical applications. Various broad research programs and innovative research infrastructures, as well as new professorships and junior research groups and training opportunities for clinician scientists and medical scientists have been established at DKTK.

However, appropriate structures, and expert workforce and flexible funds for the next stage of the translational research continuum, i.e., early‐phase clinical trials, continued to be a major bottleneck in Germany. For this reason, the National Center for Tumor Diseases (NCT) was founded as a joint initiative of DKFZ and selected UMCs to institutionally link patient‐oriented clinical cancer research with high‐level multidisciplinary cancer care. The NCT concept is to establish a long‐term and powerful clinical research platform as a branch of the DKFZ in the already existing CCCs of the UMCs, thereby generating strong synergies and leverage effects. All local NCT sites develop specific profiles and are part of the overarching ‘One NCT’ strategy and governance, which enhances critical mass and comprehensiveness. The first NCT pilot site was established in Heidelberg in 2003, and a second pilot site followed in Dresden in 2014. In the coming years, based on an internationally peer‐reviewed selection process, the NCT will be expanded to six sites involving 11 UMCs. This NCT expansion is an important strategic component of the ‘German National Decade against Cancer’ launched by the federal government in 2019. The two existing and the four new NCT sites will significantly increase the number and quality of IITs in Germany and improve patient access to innovative early‐phase clinical trials nationwide.

Adopting the successful model of CCCs (i.e., multidisciplinarity and multiprofessional approaches, a translational continuum from fundamental to implementation research, involvement of all stakeholders, integration of research with high‐level application/care, education and outreach), the DKFZ is currently establishing a National Center for Cancer Prevention in Heidelberg in a strategic partnership with the German Cancer Aid. This endeavor will integrate basic and applied‐to‐humans prevention research laboratories, an outpatient prevention clinic, an education and training center and structures to address national outreach under one roof.

The research landscape in Germany described above encompasses the entire translational continuum and is based on the successful implementation of sustainable national networks, long‐term institutional funding, the linkage of cancer research and cancer care/prevention, and the creation of structures for the training of basic, medical and clinician/clinical scientists.

**Structuring Cancer Activities in Portugal** was presented by **Rui Henrique**, Porto Comprehensive Cancer Centre Raquel Seruca, Porto, Portugal.

Portugal has 10 300 000 inhabitants, 50% of which are over 50 years, with an average life expectancy of 83 years for women and 77 years for men. Cancer incidence is rising [2019: 562.1 cases per 100.00 inhabitants, in both genders, with male predominance (648.3 vs. 485.1) – almost 58 000 new cases], having increased by 19.3% since 2010. The profile of the most common cancers is similar to that of other western countries.

The Portuguese National Health Service (SNS) provides universal and free health care, encompassing primary care and hospitals with different levels of specialized resources. According to the last reports, 47 public hospitals are involved in providing care to cancer patients, but only nine have all the core specialities to autonomously manage cancer diseases. About 80% of all cancer cases are diagnosed and managed by 19 institutions (> 1000 patients per year in each). Referral networks have been defined according to medical speciality, resulting in care fragmentation and the need for coordination among key disciplines. Considering the evolving healthcare provision scenario in Portugal, with a flourishing private sector, definition, and implementation of a nationwide strategy, involving all stakeholders and considering the whole installed capacity of the system, is needed.

Considering the three existing Oncology Institutes (Lisbon, Coimbra, Porto), all accredited by OECI, a nationwide Cancer Care Network should have these three institutions as main nodes, roughly covering the South, Center, and North regions. These should have the characteristic of CCCs, integrating high‐quality and complex care with education/training and basic, pre‐clinical/translational and clinical research. IPO Porto (Instituto Português de Oncologia do Porto) is an OECI‐accredited CCC; the rest are OECI‐accredited Clinical Cancer Centers. These cancer centers should coordinate cancer‐related activities at the regional level, with a definition of guidelines for levels of care at each node of the network, encompassing from highly differentiated institutions (e.g., university hospitals) to regional hospitals and primary care units. Developing these Comprehensive Cancer Care Infrastructures, taking patient pathways as the backbone, is key to ensuring multi‐directional collaboration within the network. This type of organization should be promoted by educational/training events as well as periodic visits and stays of professionals (physicians, nurses, technicians) across the different levels of care in the network. Moreover, standardized diagnostic and staging procedures will allow for more effective use of equipment and facilities, promoting large‐scale effectiveness across the network. The latter will also foster research at various levels, from identifying patients for observational and clinical studies to outcomes research, as updated patient information will be easily available. Furthermore, this type of organization will facilitate new interventions, from testing novel screening/early detection strategies to further treatment and follow‐up procedures, in a standardized fashion, providing reliable and high‐quality data that enables real‐world data studies. Finally, active engagement in European‐level networks is mandatory to ensure that every citizen has access to the best care, irrespective of their region of origin. Notwithstanding the challenges of building such a network in Portugal, IPO Porto has been involved since 2013 in several initiatives aiming to implement specialized cancer care in African Countries with Official Portuguese Language (PALOP). The OncoPalop project, resulting from the cooperation between governmental and non‐governmental entities, universities and hospitals from multiple countries, supports building infrastructures, and training healthcare professionals to fight cancer in Africa. Based on establishing solid and effective ties between health care professionals, this project will help those countries to deal with the expected steep rise in cancer cases over the next two decades.

**Improving access to cancer care in India** was presented by **Mammen Chandy**, Tata Medical Center, India.

There is a significant disparity in Cancer Care in India, and the main reasons for this are:

- Unequal distribution of Resources
- Education and Social factors
- Access

Better access is needed because of the gap between the available infrastructure and the demand, with few tertiary care centers and even fewer secondary and primary centers. This limits the availability of diagnostics, precision medicine, radiotherapy, palliative care and prevention, particularly for 70% of India's population with limited resources. India spends only 2.2% of its 400 billion‐dollar GDP on health, and the disparity between the rich and the poor is widening, with the top 5% owning 63% of India's wealth. Three percent of wealth tax on the total wealth of India's billionaires can fund the national health mission of India for 5 years [28].

Thus, India's problem is not the lack of resources to make a quantum improvement in cancer care and health care in general but the gross inequality in the distribution of wealth. Justice and equity are required, and we would do well to remember the words of Fred Kaan Hymm, writer for the WCC (World Council of Churches): ‘For the healing of the nations, Lord, we pray with one accord; for a just and equal sharing of the things that earth affords.’

However, it would not be possible to provide all of the therapies modern precision medicine has made available for cancer to all of India's 1.4 billion population. Therefore, a cafeteria approach may be possible based on the resources available for the individual patient. At the same time, we strive to provide the gold standard of care for all our people [29].

The magnitude of the Problem. The annual incidence of new cancer cases in India is 1.3 million, with cancers of the mouth, lung, and GU tract being the commonest in men and breast, cervix and ovary being common in women. With the population pyramid gradually becoming inverted, the proportion of older persons is growing, and India will face increasing demands for cancer services.

India has several strengths for providing better cancer care for its population, including good quality tertiary cancer centers, availability of reliable cytogenetics/FISH/next‐generation sequencing, and low‐cost generic drugs manufactured in India at 1/10th the cost of drug prices in the Western world. Importantly, the country has a strong base of educational institutions and a modern network of information technology.

India needs to develop a strong cancer prevention program emphasizing reducing tobacco use and making available analgesics for pain control in a well‐organized palliative care system.

It is certainly possible for India to close the gap and move toward equity in cancer care, prevention and treatment if only we ensure the distribution of wealth with the political will to ensure that the country's resources are used wisely for the good of its people.

**Structuring cancer activities toward personalized/ precision cancer medicine in Africa** was presented by **M. Iqbal Parker**, University of Cape Town and African Academy of Sciences, South Africa.

Our health is determined by our inherited genetic differences combined with our lifestyles and other environmental factors. Personalized medicine is a change from the ‘one size fits all’ approach to the treatment and care of patients with new approaches to better manage their health and targeted therapies to achieve the best outcomes in managing the disease.

Excellent results have been obtained in many populations where there are genomic databases that reflect the natural diversity in those populations. These have led to discoveries that made clinical trials and medical care more successful for participants with these genetic backgrounds.

Human genomic databases are heavily skewed toward European populations, so there is an underrepresentation of certain ethnic groups in these databases. This bias will continue contributing to unequal precision/personal medicine success rates.

It is of note that:

- African genome data comprises less than 2% of the human genome database.
- African populations are genetically the most diverse of all populations.
- The Human Genome Project reference genome lacks African ancestral variants.
- A study involving 426 genomes across 15 African countries using the Infinium H3Africa Consortium Genotyping chip revealed 3 million unknown variants.

The leading cause of death in Africa is due to neonatal diseases, followed by lower respiratory infections, diarrheal diseases, HIV/AIDS, heart disease, stroke, malaria, tuberculosis, road injuries, liver disease, and cancer, yet the emphasis is placed largely on HIV/AIDS, malaria and tuberculosis.

The way forward should focus on the following:

- Increased TRUE global south–south and north–south collaboration.
- Increased commitment by African governments to improved healthcare provision.
- Increased spending on human capacity development.
- Acquiring Infrastructures.
- Good reliable biobanks.

**Structuring cancer activities toward personalized/ precision cancer medicine in Australia** was presented by **David Thomas**, University of New South Wales, Sydney, Australia.

Cancer recently overtook cardiovascular disease as the leading killer of men in high‐income countries. Over the centuries, cancer has evolved through three fundamental conceptual stages driven by utility. The first stage was a macroscopic classification system based on the primary sites of cancer development. Still in use today, anatomy defines the fundamental structure of clinical management: lung clinics, GI clinics, for example. This is because specific sites require dedicated training and expertise, and surgery remains critical to cure most solid cancers. This classification failed some cancer types, which are not defined by their site of origin: for example, Ewing sarcoma. Partially addressing this, the second classification arose in the 1850s, determined by the microscope. The critical advance here was the cellular concept of cancer. Over many decades, a histologic concept of cancer has led to classifications with major impact, especially in relation to cancers of unknown lineage, like Ewing sarcoma. The microscopic appearance of Ewing sarcoma (small round blue cells) was empirically associated with the property of wide dissemination (making surgery relatively ineffective on its own) and sensitivity to cytotoxic therapy. Generally, the associations between microscopic morphology, and eventually immunohistochemical techniques, and tumor behavior has been extremely useful. Consider alone the utility of distinguishing between small cell and non‐small cell lung cancer, collectively the leading causes of cancer death worldwide. A molecular or scientific view of cancer drives the third conceptual stage. As a genetic and cellular disease, over the past 20 years, cancer has been revolutionized by genomics, molecular and cell biology, underpinning rational drug development. Although genomics has yet to enter routine clinical practice, the pharmaceutical industry years ago committed to a molecular framework for drug development with impressive success. This third stage of the molecular concept of cancer can be considered analogous to the enlightenment, ushering in an era in which a scientific framework radically advances our ability to improve health outcomes for cancer patients. However, systemic factors now present the major barrier to fully realizing the benefits of science. These barriers relate to the models for drug development in which the public sector and industry are separated and often adversarial. Drug development costs may exceed US$3B, while the fraction of GDP spent on health is rising in all countries globally. This has the potential to result in health disparities which must be addressed to achieve the full benefits of science to mankind.

**Structuring cancer activities toward personalized/precision cancer medicine in China** was presented by **Huanming Yang and Frederick Charles Dubee** Beijing Genomics Institute (BGI), Shenzhen, China.

China has much to learn and is committed to learning and sharing scientific knowledge while steadfastly refraining from imposing its views. There is a sacred and indivisible relationship between science and ethics that is at the core of science and vital for every scientist. While fully acknowledging the importance of therapeutics and care, this presentation focused on the reduction of the incidences of cancer and the reduction in the severity of unavoidable incidence as key factors in decreasing the inequities in cancer therapeutics and care.

Explorations of the question of What is Life, the discovery of DNA double helix, the sequencing of the Human Genome, and the advances in computing and sequencing combined with the accelerated development of public health have given humanity the possibility of starting with confidence at least 30% and probably closer to 50% of all cancers are preventable.

While public health allows us to identify and understand health and cancer‐causing threats to entire populations and suggest policy measures to reduce or eliminate risk, the developments in genomics, screening, and testing allow us to zero on the dangers or the actuality of potential problems, pre‐problems, or problems in earliest stages of development and provide state‐of‐the‐art information on which sound decisions can be discussed and made.

Improving our understanding of risks at the population and individual level and early detection of potential or nascent problems permits taking action at stages where the impact on the patient and family is at or near the lowest point.

China, through programs like ‘Healthy China 2030’, the intensive developments in genomics supported by an array of social, scientific, and industrial efforts form an integrated community focused on the reduction of the incidence of cancer cases.

To broaden awareness and in recognition of the efforts of the PAS and the EACS, the China‐based International Conference on Genomics and its 18th Annual meeting (ICG‐18) has initiated a project that focuses on sharing the message of the PAS and EACS to scientists, researchers, healthcare professionals, and policymakers first in China and subsequently in the Global South.

### 5.2 Discussion and conclusions

The session discussed several examples of structuring cancer activities in EU member states by the OECI, in Portugal and beyond (South Africa, India, Australia, and China). The situation in various countries and regions is quite different regarding healthcare systems, structures, creation of networks and unmet needs.

In Europe, CCCs are at different stages of maturation in the various regions (North‐West, South, and East Europe). The OECI has spearheaded the establishment and accreditation process of CCCs and is very active in developing such structures in the region with the greatest unmet need Central‐Eastern Europe (CEE). CCCs thrive on integrating care and research, development of translational research, education and training. Integrative care and multidisciplinary tumor boards are core elements in such functioning. To facilitate the creation of these structures, the principle of twinning programs between established CCCs and developing CCCs was stressed. Germany has seen three major developmental projects to achieve this over the last 15 years. The CCC program of the Germain Cancer Aid and the Translational Research network DKTK, coordinated by the German Cancer Research Institute (DKFZ), are key programs that have been very successful. The completion of these programs led to the National Center for Tumor Diseases (NCT) program; a federal initiative coordinated by DKFZ. It is a prime example of how well‐structured and well‐financed programs can completely transform the landscape of Cancer Care and Integrated Cancer Research. Interestingly Portugal is working on a similar approach, as presented for the Porto Cancer Research Institute development.

Australia reported a well‐structured centralized public‐private enterprise to advance molecular testing to facilitate the best choice for the treatment of cancers. Like in the EU, a centralized public health care system with large referral hospitals provides opportunities to establish this. The importance of further integration to advance cancer care was stressed.

Similarly, China reported a strong program on reduction on cancer incidence and development of PCM. A key role in this development is the program spearheaded by the Beijing Genomics Institute (BGI) in Shenzhen and similar sites in all major cities of China. Remarkable progress over the last decade led to a significant increase in access to new generations of targeted and immunotherapeutic drugs.

The situation in India is one of major challenges. Lack of healthcare infrastructures in most rural regions and even in large cities, unequal access to care, unequal distribution of wealth, and a great need for education and training define great unmet needs. Healthcare offers services that range from excellent to rudimentary/non‐existent creating profound unequal access to care in general and cancer care in particular.

In Africa, one encounters similar problems. Interestingly we learned that African genome data comprises less than 2% of the human genome database. African populations appear genetically the most diverse of all people. The Human Genome Project reference genome lacks African ancestral variants. A study involving 426 genomes across 15 African countries using the Infinium H3Africa Consortium Genotyping chip revealed 3 million unknown variants. When one adds to that the lack of health care infrastructures and unequal distribution of wealth and multiple regions with pronounced poverty, one is facing a tremendous challenge. Unequal access to even the most basic care, let alone advanced cancer care is a major concern.

Overall, unequal distribution of wealth, healthcare structures and education determine the current situation in our world. Joint programing, networking and development programs are urgently needed to tackle the current unmet needs.

## 6 Session 3: How to exploit innovation as the driver to reduce inequalities

### 6.1 Chair Anton Berns, Netherlands Cancer Institute, Amsterdam, The Netherlands

**Comprehensive Cancer Centres: Achieving Critical Mass, sharing advanced infrastructures. EACS recommendations** was presented by **Anton Berns**, the Netherlands Cancer Institute, Amsterdam, The Netherlands.

Cancer Research Infrastructures are of critical importance for offering cancer patients state‐of‐the‐art treatments, providing optimal environments for basic, translational, prevention, and clinical research as well as to assess the effectiveness of new treatments and the associated health‐related quality‐of‐life of cancer patients. In combination with EU‐wide registries, biobanks and data repositories such infrastructure can boost research throughout the cancer research continuum and concomitantly the quality of cancer care in European member states. The MoC and EBCP should foster the establishments of such infrastructures. There is a need for infrastructures for basic and translational research, for clinical trials, and for outcome research. Furthermore, it is essential that the long‐term sustainability of such infrastructural facilities is secured. The CCC combining basic‐translational and clinical research should serve as the cornerstone of the cancer research continuum and provide first‐rate care to cancer patient. It should have sufficient critical mass for an interactive research environment with core facilities offering the necessary access to fully up‐to‐date, ‘omics’, cell‐based screening capacity, molecular pathology with AI‐assisted pattern recognition, pharmacology, bioinformatics, and support to conduct data‐rich clinical trials. Patients need to be put in the center with access to state‐of‐the‐art treatments and overall care with quality of life as guiding principle. These CCCs will also be well‐positioned to generate the high‐quality data needed to assess the effectiveness of treatments and thereby for conducting outcomes research. CCCs come in different flavors, e.g., the CCCs accredited by OECI with a well‐integrated (pre)clinical trajectory and late translational research, whereas the EACS designation of excellence requirements [30] for CCCs demands international competitiveness in the complete trajectory from basic research to late translational research and innovative clinical trials. CCCs also should closely interact with nearby hospitals, universities and research institutes to enable optimal treatment of cancer patients in the regions served by these hospitals. This will also facilitate recruitment of patients in clinical trials.

Furthermore, CCCs should form modestly‐sized networks to further expand their capacity to recruit patients, to support education of the next generation of cancer scientists and clinicians and assist in the establishment of CCCs in less‐privileged regions in Europe (twinning arrangements). There is also a need for collaborating networks – not necessarily exclusively consisting of only CCCs‐ that focus on clinical trials, on prevention research and outcomes research.

A good example of such a network is Cancer Core Europe (CCE) [31] in which 7 CCCs/Universities work closely together resulting in a high volume of clinical activity (25–300 K patients treated/year) with highly developed research infrastructures. This results in (a) optimal recruitment capacity through joint Clinical Trial Operations, (b) flexible trial designs to evaluate new treatment modalities, (c) critical mass to study challenging cancer types and address cancer complexity, (d) a virtual data center: detailed high‐quality data: new insights, (e) standardized patient records permitting Outcomes Research, (f) standardized regulatory legal procedures, centralized by a dedicated CCE Task Force, (g) education and training of next‐generation Translational–Clinical Scientists, and (h) a boost in quality by mutual engagement.

The benefits of such arrangements are evident. However, it is a major effort to establish these networks and an even greater challenge to secure their sustainability. Here the EU‐programs aimed at providing state‐of‐the‐art cancer care and cancer prevention measures to EU citizens could be of enormous help for their establishment and providing incentives for their sustainability.

**Inequalities in cancer research – improved science with improved outreach** was presented by **Peter Nagy**, National Institute of Oncology, Budapest, Hungary.

Cancer poses a great burden on society and citizens, and although Europe provides outstanding cancer care, notable disparities exist between European countries and even within the countries. This was corroborated by our recently published research which underscores the continued existence of an East–West divide in life expectancy across the EU, evidenced by benchmarking cancer mortality in founding members vs. countries which accessed the EU after 2004 [32].

In order to reach the aim of 70% average 10‐year survival for all European cancer patients by 2035 (70:35 aim), socio‐economic inequalities in cancer need to be reduced as well as inequalities in cancer research need to be addressed in a pan‐European manner. While the difference in incidence in all cancer cases between the Western and Central and Eastern European (CEE) countries is not significant, major differences exists between the aforementioned regions when it comes to mortality. This needs to be addressed both in the MoC and in the EBCP programs. An important recently published paper by IARC demonstrated that although mortality rates are higher among the lower‐educated individuals for nearly all cancer types in all EU countries, the differences in survival between lower and higher educated populations are much higher in the CEE region as compared to the Western member states [13]. It could be generally concluded that the higher rates in overall mortality in the CEE region can be associated with the worse prognoses among the lower educated populations with worse socioeconomical status. Potential causes of these inequalities include individual and collective behaviors, customs and social interactions linked to the exposure to cancer risk factors, availability and access to early diagnosis and screening programs, availability and access to effective treatments, as well as research facilities and research activities.

CCCs play a key role in cancer care and we recently concluded that patients treated in more research‐active centers have better outcomes [24]. While numerous accredited CCCs are present across Western Europe, in the CEE region currently only Hungary has a CCC, the National Institute of Oncology. While overall the European continent is a global leader in cancer discovery science, major differences within the continent exist in cancer research outputs specifically when looking at the gap between the EU13 (Member States that joined the EU in 2004 and after) and EU28 (EU up until 2020) countries in terms of outputs of biomedical research papers as well as cancer research papers [27].

Work Package 6 in the 4. UNCAN.EU project entitled ‘Inequalities in cancer research – improved science with improved outreach’ is designed to address inequalities in cancer research across the European region with a special focus on CEE. The work is led by the National Institute of Oncology in Hungary and co‐lead by the Oncode Institute (the Netherlands). The major goal of this activity is to identify mechanisms that may boost cancer research and innovation potential in lower income regions across Europe while promoting technology transfer and interaction with private companies. The main tasks of this Work Package are focusing on the following areas: (a) To reduce inequalities in cancer research and innovation by benchmarking existing research infrastructures and networks across Europe through a survey of cancer research institutes using indicators developed by OECI and adapted to 4.UNCAN.eu objectives (infrastructures, research and innovation agenda, open access data, collected material, national and regional resources, identified limitations, key research outputs) to be crossed with a literature survey of scientific outcomes. (b) To generate rules that promote twinning programs by evaluating the outcome and challenges in existing twinning programs between CCCs in Western and Eastern Europe and by formulating recommendations to promote the process of ‘matching’ suitable complementary partners in 4.UNCAN.eu programs. (c) Identify opportunities for technology transfer and industry collaboration with a special focus on regional gaps to boost innovation and research potential across Europe. (d) To identify specificities of cancers across member states that may drive research programs. The tasks are led by (a) the National Institute of Oncology and OECI, (b) DKFZ, (c) Oncode Institute in the Netherlands and (d) IARC with the participation of other international organizations.

In conclusion, there are remarkable inequalities in cancer incidence and mortality within Europe, and socioeconomic inequalities are represented in cancer statistics. In order to achieve the ‘70:35’ aim, these disparities should be addressed urgently. Since better cancer research leads to better cancer care in MS, there is a need to develop a pan‐EU research and innovation plan, which addresses inequalities and involves the improvement of research infrastructures within the CEE region. In addition, cancer prevention should be a priority not only in CEE, but across the whole European continent. The 4.UNCAN.eu initiative is dedicated to set the stage to address inequalities in cancer research across Europe.

**Structuring sharing of infrastructures and patients for precision cancer medicine clinical trials in Norway** was presented by **Åslaug Helland**, Institute of Clinical Medicine, Oslo, Norway.

In 2018–2019, the clinical environment raised awareness to new cancer drugs being developed that required molecular testing not yet available in the public health care system in Norway. Processes were initiated, both bottom‐up and top‐down, to implement precision cancer medicine. In addition, connections were made with similar efforts in the Nordic countries and in the Netherlands, and a public‐private partnership was established [33, 34].

Since then, advanced molecular diagnostics analyzing > 500 genes on DNA/RNA level have been established as a reimbursed service to patients with advanced malignancies. Since the initiation 2 years ago, we have been scaling the service, from one hospital serving the whole country to four hospitals today. In parallel with the development of the diagnostic pipeline, a molecular tumor board has been established, involving clinicians from all hospitals treating cancer patients.

April 1, 2021, a national clinical trial was opened for inclusion, called IMPRESS‐Norway (Improving public cancer care by implementing precision medicine in Norway). This is an investigator initiated clinical trial for patients with advanced disease no longer benefiting standard treatment, collaborating with the pharma industry who provides drugs. It is a study similar to the DRUP‐trial in the Netherlands, and also as the other Nordic trials.

In IMPRESS‐Norway, we started with eight different drugs in April 2021, and now have 22 drugs available for our patients. The drugs are used outside of current indications.

All hospitals with a cancer department are participating in the trial. The patients are screening using the advanced molecular diagnostics, discussed in the molecular tumor board and if a biomarker matches one of the drugs, a patient can be offered treatment in IMPRESS‐Norway. Several patients have also been referred to other ongoing studies, and to early access programs, resulting in new treatment options offered one third of the patients in total. Through the clinical study, we collect biological material for whole genome sequencing, analyses of circulating tumor DNA etc, providing data and material for research groups throughout the country.

Through this work, Norwegian cancer patients have gained access to advances molecular diagnostics, and also a trial providing possible treatment for the patients. Some pharma companies and diagnostics companies have supported the efforts, and we have a platform for discussions between the public and the private partners.

Two EU‐funded projects are now aiming at developing similar structured trials in more European countries, including countries in the Baltic region, central and southern Europe in addition. Precision cancer medicine can provide treatment to very small patient groups, and collaboration is necessary to gain insight and collect data also in these small groups of patients.

**Cancer Prevention Europe, a consortium for cancer prevention research** was presented by Joachim Schüz*, IARC/WHO, Lyon, France.

Cancer Prevention Europe was established in 2017 to complement excellence networks of cancer care, with the aim to bring together key institutions for cancer prevention research of Europe [35]. Cancer Prevention Europe originated in a collective recognition that cancer prevention in Europe is fragmented and not fit for purpose, as the vast majority of Europe shares the same risk factors for cancer. An overall strategy was therefore desperately needed and indeed at present primary, secondary, and tertiary cancer prevention have higher visibility than ever before. At the same time, it was decided to update the European Code against Cancer, a key cancer prevention tool first developed almost 40 years ago, to for the first time not only give evidence‐based recommendations to individuals of how to reduce their risk of cancer, but include health care workers and enablers as specific target groups, link to existing cancer prevention policies, and find synergies with other non‐communicable disease [36, 37]. Numbers of new cancer patients are rising all over the world, and Europe is no exception to this trend [38]. With an estimated 4.4 million new patients in Europe in 2020 (United Nations definition of Europe), a rise to 5.2 million is predicted for the year 2040, resulting in about 100 million new patients over the next 20 years. Not even the wealthiest countries can therefore only rely on treatment, as this is not only a question of economic resources but especially also human resources for treatment and after‐care. This increase is not a worst‐case scenario but the most realistic one given the expected continued increase in life expectancy in Europe, with the risk of cancer also increasing with increasing age. This can be best illustrated for the case of lung cancer in men, for which tobacco is the main risk factor explaining 80–90% of all those therefore preventable cancers: when accounting for the aging effect, the (age‐standardized) incidence rate of lung cancer in men decreased by more than one third in the last 40 years in the Nordic countries due to successful prevention action against tobacco use, but when looking at the numbers of newly diagnosed male lung cancer patients every year, due to the increase in the number of men at higher risk for lung cancer because of higher age, this has never been higher than for 2020. The tobacco‐cancer epidemic is far from over and like for smoking prevention efforts for healthier diet, more physical activity, healthy body weight, and no alcohol drinking have to be intensified. It is those lifestyle risk factors that also mainly explain the social inequalities in cancer that are observed across Europe [13]. Even though the magnitude of inequalities in cancer mortality varies by country and over time, there are also similarities, and one remarkable observation is that mortality rates vary much less among the better‐educated. Cancer Prevention Europe is tackling improvements in cancer prevention through research at many levels. First, research into identification of causes of cancer and better understanding of mechanisms of cancer remains a high priority, as causes of cancer are only known for about 50% of the total cancer burden [38]. Second, intervention research is important to establish the most successful interventions for cost‐effective prevention. Third, implementation research is important as even when successful interventions are known, it is not that all size fits all, and different population groups (by country, by age, by gender, and – obviously – socioeconomic position) will require adaptation of those interventions.

*Disclaimer.

Where authors are identified as personnel of the International Agency for Research on Cancer/World Health Organization, the authors alone are responsible for the views expressed in this article; these views do not necessarily represent the decisions, policy or views of the International Agency for Research on Cancer/World Health Organization.

**Chien‐Jen Chen**, Academia Sinica, Taipei, Taiwan, delivered a presentation on **Prevention, early detection and treatment of cancers caused by bacteria and viruses in the Asia‐Pacific region**. No abstract [39].

**How will the EU‐project UNCAN prioritize and implement innovations from basic/preclinical research into clinical/prevention research?** presented by **Eric Solary**, Gustave Roussy Cancer Campus Grand Paris, Villejuif, France.

Two convergent novel initiatives of the EU – in health, EBCP and, in research and innovation, the Horizon Europe MoC – spurred the creation of a European Federated Cancer Research Data Hub to better UNderstand CANcer (UNCAN.eu). The global ambition of UNCAN.eu is to collect research data, patient health data, and any other relevant data at an unprecedented scale to gain a new and deeper understanding of cancer mechanisms. The objective of the coordination and support action named ‘4.UNCAN.eu’, which was launched in September 2022, is to deliver a strategy roadmap for the creation and implementation of UNCAN.eu at the end of November 2023.

The European Federated Cancer Research Data Hub will collect clinical and real‐life data together with images and omics data. A complementary ambition is to collect data from cancer research models. UNCAN.eu could include a federated data hub for all the patient data under GDPR and a centralized data hub for non‐GDPR data. The federated organization will require the creation of national or regional cancer research data hubs subsequently gathered in the European organization that will be managed according to the FAIR (Findable, Accessible, Interoperable, Reusable) principles.

To initiate the data hub, the blueprint will include use cases in the form of cancer research challenges to tackle at the European level. These challenges will collect or generate cancer research data that will start feeding the data hub following standardization rules. Challenges are defined by European experts through a bottom‐up process in six predefined intervention areas. Meanwhile, 4.UNCAN.eu is mapping the existing areas of expertise in data collection across European countries as well as the optimal European research infrastructures that could contribute to UNCAN.eu objectives, including a partner infrastructure to operate the Federated Cancer Research Data Hub. The strategic roadmap includes the creation of a high level strategic and scientific committee that will guide the collection and use of cancer research data, identify new potential resources that could enrich the field, and propose to the EC the new challenges to address at the supranational level in cancer research.

A governance model is prepared together with a business plan that will define the needs for a sustainable organization and identify the most appropriate legal entity in order to manage UNCAN.eu platform. Two additional objectives are addressing potential sources of inequalities. The first one is the role of patients, patient advocates and citizens in cancer research, including the governance of the proposed platform and its strategic development. The other one is the differential ability of MS and Regions across Europe to generate, collect and share appropriate high‐quality data to address the largest cancer research challenges that we may face in the coming decades. This later issue will require to boost the potential of lower income regions in order to ensure complete EU coverage of data collection and expertise along the time. Twinning programs and the creation of incentives to create the needed national data hubs are part of the tools identified to reduce inequalities in cancer research resources in Europe.

**The outcomes research needed for health economics research of therapeutics and Prevention – how to achieve cost‐effectiveness** was presented by **Bengt Jönsson**, Stockholm School of Economics, Stockholm, Sweden.

All activities in the cancer control continuum use resources such as manpower, capital, medicines and other inputs to produce outcomes of different kinds. The use of resources can be aggregated to a measure of total costs using prices for defined resource units. For comparisons of costs and outcome you need an aggregated outcome measure that be used for all types of interventions and for all patients. Quality‐adjusted life years (QALY) is a measure that combines the two most important outcomes for cancer patients, survival and quality of life [40]. One advantage of this measure is that it can be used for both epidemiological studies as a measure of the burden of disease and its variations between populations and over time, and as an outcome measure in economic evaluations aimed at informing decisions about choice between relevant alternatives for using scarce resources. Medical research produces an increasing number of alternatives, with different costs and outcomes, for using resources during the cancer continuum. Variations in income per capita between countries, and the resulting difference in what can be spent on cancer care, is the major reason behind inequalities in access to high quality cancer care. But it is not the only reason, which is illustrated by similar differences within countries. Studies that provide information on cost‐effectiveness of different options for controlling cancer will be of value for all countries and should be undertaken in international collaboration with the majority of funding from the richer countries.

The same evidence about cost‐effectiveness should be required for all uses of resources for prevention, early detection, surgery, radiotherapy or new cancer medicines, but the later needs a specific attention due to the rapid increase in alternatives and costs [41]. New cancer medicines come to the market with evidence that they are safe and effective, but the available information is not enough for assessment of outcome and cost‐effectiveness. There is great uncertainty about the magnitude of improvement of outcome in clinical practice over available alternatives. Still the most common decision is acceptance after negotiations with a discount on the price. The most relevant decision would be coverage with evidence development and payment linked to the observed outcomes. But in most countries, there is neither funding nor competence for undertaking such studies. CCCs in collaboration could build the competence and be a vehicle for designing and undertaking such studies.

### 6.2 Discussion and conclusions

The take home lessons from this session can be summarized as follows. To prevent that the cancer burden will overwhelm the health care system and deprive citizens of state‐of‐the‐art cancer care, the following initiatives should be taken:

First of all, we must invest in research covering the complete cancer research continuum with specific emphasis on primary and secondary prevention as that it can substantially diminish the number of patients with uncurable cancers. Furthermore, we have to realize that we simply will not have the personnel nor the financial resources to provide high‐quality treatments to patients if both their number and the costs of therapies continue to increase, with a specific concern regarding the skyrocketing cost of anti‐cancer medicines [42]. Both quality‐of‐life and cost‐effectiveness should serve as the guiding principles. This will require infrastructures in which best‐practices can be developed and tested. Establishment of CCCs throughout Europe and elsewhere wherever possible (at least one per 5 million citizens) will be critical as well as their benchmarking to promote quality. A substantial fraction should conduct innovative research that covers the complete research continuum from basic research to implementation and cost‐effectiveness research as a route to optimal cancer care. CCCs should reach‐out to local hospitals to assure that all patients in their region have access to the best treatments with a central role for the patient. CCCs need to establish networks to learn from each other and boost quality and secure access to sufficient patients to enable efficient data‐rich PCM clinical trials. The advanced CCCs should take the lead in educating the next generation of cancer researchers and clinicians. Twinning of these CCCs with CCCs in less developed regions can accelerate the development of high‐quality centers in the latter regions. We already witness the benefits of such networks. Examples are CCE, the collaborating centers in Germany but also in Scandinavian countries in which hospitals started to closely collaborate with installment of joined tumor boards and introduction of sophisticated clinical trials designs (e.g., IMPRESS with a set‐up similar to the DRUP trial in The Netherlands). Support from the MoC to specifically facilitate the establishments of these infrastructures is critical and deserves more emphasis than it currently receives. Its promotion together with the establishment of an EU‐wide Data Hub in which relevant patient information and omics and imaging data sets can be shared and for which a blueprint is being designed within the UNCAN program is strongly recommended. Thereby it is crucial to put emphasis on prevention and implementation research e.g., by further facilitating and expanding initiatives as taken by Cancer Prevention Europe. We need to better define the causes of cancer, individual predisposing factors, design prevention trials based on the knowledge of how cancer develops and put more emphasis on implementing effective treatment. We also currently fail to act on well‐known environmental and lifestyle risk factors. In this regard vaccination against virally induced cancers tell an important story. HPV vaccination is one example with evident results, although many more individuals must be reached to achieve the full effect. Another example is the substantial reduction in liver and stomach cancer in Taiwan by well‐organized vaccination programs against hepatitis B/C and treatment of Heliobacter Pylori infection, as was reported by Chien‐Jen Chen. These investments in basic, translational and clinical cancer research must go hand in hand with outcomes research. Measuring parameters such as adjusted quality of life years can assess the added value of new treatments in daily practice and their cost‐effectiveness within the overall health‐care system. It is clear what needs to be done. Now we have to do it!

## 7 Concluding session

### 7.1 Chairs: Joachim von Braun, president PAS, and Michael Baumann, president EACS

**Ingemar Ernberg** from the Karolinska Institutet, Stockholm, Sweden, highlighted **How** **education will play an instrumental role in decreasing inequalities in cancer therapy, care and prevention**.

Today, there is a need for educational actions to decrease inequalities in cancer therapy, care and prevention across Europe and worldwide [43]. The needs range from informing the general public about cancer risks and possible lifestyle improvements to acting on them to reach out with best practices to patients, their close relatives, healthcare staff who treat cancer and scientists.

One major reason for the conspicuous differences in cancer incidence and mortality across Europe is that available knowledge and practices do not reach out to all. Although it is hard to estimate more precisely how much of the outcome inequalities are due to variable access to knowledge (e.g., risk factors to act upon) compared to variations in investments in health care, educational efforts will be a cheap, cost‐effective and fast, compared to the challenging task to reform health care systems.

Today, education related to the cancer problem is fragmented, with many action areas and actors. Advances can be achieved through relatively small measures of collaboration in education between European states, particularly if efficiently organized: implementation of the right educational activity at the right level. Recent developments, including the MoC, the EBCP and the CCCs in many EU countries, offer new and powerful possibilities to deal with unmet educational needs in an efficient and structured effort [44, 45, 46].

Educational and training activities should target the general public (dissemination) in primary cancer prevention and the next generation of cancer researchers in basic and clinical research all over Europe. The teaching faculties of universities and medical and nursing schools are responsible for the undergraduate professional education of health care staff at all levels as well as that of researchers. The training of physicians and scientists is somewhat harmonized across Europe according to initiatives like the Bologna process.

Multidisciplinary cancer treatment and care needs the support of educational activities directed toward the healthcare staff to deliver high‐quality, an issue primarily for the healthcare systems themselves. These increasing needs generated by new technologies and more integrated approaches have only been realized at the University Hospital level. Regional and local cancer care providers must also be involved in defining the needs.

It is more of a challenge to establish a functional clinical exchange program for physicians and other healthcare staff between European university hospitals and CCCs. Once in place, such an action will prove invaluable for enhancing collaboration, harmonization and dissemination of best practices. Small pilots have started aiming for 1 week to 3 months of exchange. Financing and language barriers are current obstacles.

Several European organizations such as the European Society for Medical Oncology (ESMO), the International Society of Pediatric Oncology (SIPO), the European School of Oncology (ESO), the European Molecular Biology Organization (EMBO), the Federation of European Biochemical Societies (FEBS) and Cancer Core Europe (CCE) offer continued postgraduate education to clinical and research professionals via congresses, courses, workshops and publications. Thus, there is reasonably good coverage of professional postgraduate training for clinicians and researchers. However, to improve the dissemination and accessibility of these extensive activities, some coordination would be helpful, including one single internet site surveilling all the upcoming educational opportunities, may be linked to the MoC's home page?

The seven CCCs of CCE have launched a three‐year advanced educational program (designated TRYTRAC) for training a coming generation of clinical/research leaders, demonstrating the important role that the CCCs should have in building competence at a high level and with international (European) networking in focus [30, 47]. The mission approach to cancer is expected to enhance the exchange of researchers within Europe further and prioritize collaborations between Western/Central and Eastern European countries.

The advantage – and necessity – of involving patients and relatives in the whole spectrum of improved cancer treatment, from patient‐reported data to the design of clinical research and trials, is now rapidly and broadly acknowledged. This will need new forms of educational activities not yet fully elaborated.

There is a broad consensus that prevention and lifestyle changes in the long term can contribute to eradicate a substantial part of the unequal cancer outcomes [46]. Although several of these lifestyle and risk factors are well known, political commitment and research are needed to implement this knowledge successfully. This has to involve public health institutions and authorities to implement information and education of the general public, most likely also requiring new forms of reaching out.

We think it would be important for the MoC and the EBCP to urgently pay attention to an all‐over strategy for education in cancer, including the different demands for different target groups and formulating ideas on how to coordinate and channel resources to avoid overlaps, duplications and misuse of educational competence.

Next, **Manuel Heitor**, University of Lisbon, Portugal, addressed **Decreasing inequalities in cancer therapeutics/care and prevention: *a policy perspective***.

**The context: *a complex transdisciplinary policy issue***.

Cancer is an increasingly relevant public health issue, and its impact is unevenly distributed, with greater relevance in the most vulnerable societies, with increasing inequalities within and among countries.

It has become critically relevant for people with low incomes, but it is also increasingly relevant in terms of ‘public understanding of science’ and is increasingly affecting the social context about the way people ‘believe in science’ [48].

It is under this context that the fight against inequalities in *cancer research* and *prevention and therapeutics/care* should be understood as a ***cultural movement*** based on a trans‐disciplinary approach that joins people at large with scientists and health professionals, as well as with artists, historians, social scientists and other academics; researchers with entrepreneurs and professionals, and students with experienced academicians in a range of research and teaching initiatives on the interface between theoretical analysis and hands‐on practices.

This brief policy contribution considers ***five points***, as follows.

I The nature of disparities in cancer prevention and care: *understanding ‘health gradients’*

Cancer prevention and therapeutics/care show important disparities between and within countries, and related inequality ‘gradients’ need to be assessed in association with other economic and income gradients. Health gradients must be better understood because they tend to be associated with different economic gradients (e.g., access to information that decreases risk factors and fosters behaviors to face risk awareness).

Under this context, cancer inequalities should be understood in a context of increasing uncertainty and unsettled minds [49], with relevant political impacts favoring extreme political parties and populist movements worldwide. In addition, if countermeasures are not taken, these inequalities will further increase disparities in access to innovations in anticancer opportunities. For example, bringing new technologies for early cancer detection using biomarkers to low and middle‐income countries is becoming increasingly important. Likewise, liquid biopsies are used to monitor cancer progression and the efficacy of therapies.

II Policy instruments: *The need for specific incentives to encourage innovation*

The analysis clearly shows that *incentives* must be considered together with *Infrastructures* and *Institutions*, as clearly stated in the Heidelberg Manifesto of October 2022 [50].

- Incentives: are always scarce and not enough, although the advancement and specialization of knowledge require additional and diversified incentives. Traditional public support of cancer science and technology can be deployed in various ways, including through *grants, procurement contracts and prizes*, individually or in combinations.
- Institutions: require autonomy and strategic capacity to guarantee a ‘cancer research continuum’. Integration with healthcare is essential, and CCCs should be responsible for orchestrating multidisciplinary cancer therapeutics/care, reaching out to areas with several million inhabitants.
- Infrastructures: require three types of critical infrastructures to address the cancer research continuum, as clearly described in the *Porto Declaration of Cancer Sciences* of 2021 [10], as follows: (a) basic and translational research; (b) clinical trials; and (c) outcome research.

But it should be clear that there is a need for specific incentives and instruments to diminish inequalities through innovation, which may include:

- Foster the use of ‘prizes’ oriented toward reducing inequalities (in addition to those based on scientific or technical merit) to complement traditional incentives for innovation;
- Foster regulatory frameworks that will impose a fair sharing of profits by pharmaceutical firms to reduce inequalities in access to drugs;
- Adopt ‘advanced market commitments’ at national and international levels (e.g., EU), under which governments commit to investing in translational research and/or guarantee reimbursement for a certain volume of a therapy that does not yet exist if market prospects are limited for some conditions.
- Explore alternative production methods and promote local production to access health technologies and medicines. Governments must support strategic projects to establish these.

III Affordability: *the critical role of international development funds*

International development funds should consider capacity building in cancer research and infrastructures for their underpinning, together with incentives for innovation and collaboration.

Although well‐equipped cancer treatment centers with expert personnel offering high‐quality cancer care are a prerequisite in all countries, we need to understand that low‐ and middle‐income countries require support and collaboration to attain substantial benefits from even relatively modest, dedicated cancer treatment centers with good diagnostics, radiotherapy, surgery, and adequate access to a subset of cancer medicines with proven effectiveness. Moreover, new treatments, like chimeric antigen receptor (CAR) T cell therapy, are changing the paradigm in hematologic malignancies, but inequities in access are enormous. The high costs of these therapies will play a role in the sustainability of many healthcare systems. Therefore, efforts are needed to address and eliminate these disparities, especially for minorities and those in low and middle‐income countries.

Complemented with legislation (including restricting access to tobacco; and differential patenting to avoid patent protection in those countries), public cancer awareness programs, screening for early detection and active prevention (HPV vaccination) may lead to substantial improvements. This requires international support for developing innovation ecosystems and policies integrating social needs, capacity building of national health systems and strengthening local research and industrial production capacity.

From a humanitarian point of view, it is important to involve all cancer patients, with specific attention to the Global South, where population growth will have a major impact in the coming decades on inequalities in access to cancer care and prevention. From an innovation point of view, international collaboration based on sharing patients, biological materials, technological resources, and competencies is necessary for optimizing research for prevention and therapeutics/care.

IV Engaging the Pharmaceutical Industry: *the need for new policy frameworks*

The pharmaceutical industry is a critical partner in the cancer research continuum and is pivotal in developing and testing new drugs. However, pharmaceutical firms should adapt their profit policies to reduce inequalities in drug access.

In addition, academia and pharma need to make better arrangements to accelerate drug development, facilitate rapid testing of single agents, evaluate combination therapies, and find ways to secure affordability for patients while permitting fair but societally acceptable returns for their contributions. The latter also holds for individual investigators starting up new companies. Reviewing patent laws to remove ineffective incentives is desirable in the long run. These may include adopting regulatory frameworks based on ‘differential patenting’ for developing countries, so treatments would not be subject to patent protection in those countries.

V Innovative and collaborative research and advanced training: *a challenge for the future*

- The fast‐rising amount of clinical and biological information requires increasingly sophisticated infrastructures and highly specialized staff to conduct research aimed at personalized/precision cancer medicine, including data handling and processing. Among many other issues, attention should consider the following:
- The ***digitalization of the cancer research continuum*** is already becoming a reality, from digital pathology to digital outcome research. International collaboration based on sharing patient data and referring patients to specialized services with the necessary resources and technological competencies is required to optimize prevention and therapeutics/care research. In addition, promoting open access to new knowledge through digital observatories of cancer research and care should receive priority.
- It is important to give priority to the establishment of a wider ***diversity of genomes in genomic databases*** than currently exploited to translate precision medicine research into practice. For example, underrepresentation of non‐European populations in genomic databases is problematic because it may miss gene‐disease relationships for which the exposure or outcome is rare in individuals of European ancestry. Furthermore, it limits the generalizability of findings from genomic research and its translation into clinical care in diverse populations.
- Improving ***research career paths*** throughout the entire research continuum has become critically important. For example, the success of international cooperation will depend on the career paths at the institutional level in each region/country. The latter will require improving recruitment, rewarding and assessment systems to increase the appreciation and value of research performance beyond scientometry, thus encouraging openness, humanism, collaboration and sharing to increase research quality and impact.
- ***Advanced training*** must promote the integration of basic, preclinical and early clinical/prevention research to improve the link between basic and clinical sciences and close collaboration within and between CCCs. Exchange programs doctoral students and for young researchers should be further expanded to secure the education of the ***next‐generation leaders***.

H.E. Msgr. **Vincenzo Paglia**, President of the Pontifical Academy for Life delivered a presentation on **Perspective from Holy See on Decreasing inequalities in cancer therapeutics/care and prevention**.

The increasing burden of cancer on society and the rapidly increasing costs of cancer for health systems require collaboration on an ambitious scale, innovating and integrating fundamental, translational, clinical, and implementation research, underpinned by supportive policy and legislation. Furthermore, today's situation is characterized by individual and often fragmented research activities and policy initiatives aimed to improve cancer prevention and control at international and national level. There is a need for better understanding of the development of cancer and of effective cancer prevention, screening programs, diagnostics, and treatments. Existing cancer guidelines are not consistently implemented across different countries, resulting in differences in standards of care and outcomes between and within States and regions. There is also a lack of understanding on the quality of life of patients during and after cancer treatment. Despite progress in cancer control through the implementation of screening programs in recent years, there is still considerable room for improvements in many countries.

One of the core values for this action should be the shared commitment to universal access to high‐quality care financed on the basis of equity and solidarity. Unhindered access to prevention and care is often under pressure within health systems broadly, and in the field of cancer in particular, due among others to widely shared pressures on limited resources. The result of this set of factors is a worldwide inequitable access to cancer prevention and timely, high‐quality diagnostics and treatment. The causes of these inequities should be analyzed and strategies should be developed to overcome them. Education has a crucial role to improve citizens' health literacy, to expertise (e.g., training for care professionals) and to research and innovation resources.

In the field of oncology, it is necessary to remember, talking about equity and justice, palliative care (including pain treatment), knowing the need to make them accessible to everyone and everywhere. Certainly knowing that these kinds of therapies are indeed accessible has an impact on quality of life even in earlier stages of illness. This is a front the Holy See is engaged on. But the Holy See is also working to encourage collaboration among researchers, health care systems, and political governance to share research projects, prevention and treatment. It also fosters attention to the most disadvantaged, geographically and economically vulnerable situations and people, to promote greater justice and solidarity. Finally, the whole perspective of the Holy See is about spreading trust and hope, for a common commitment, in fraternity and social friendship, in the formation of so many doctors, nurses, volunteers, valuing their work and the effort if NGOs in this field.

## 8 General conclusions

**Joachim von Braun and Michael Baumann** closed the conference by reminding the audience that cancer is a global problem whose incidence and outcomes adversely affect socio‐economic and political structures within and across countries. We know the world has large inequities, but we must not accept them. In 2020, the top 10% of income earners captured about 50% of the world income share. The average European has about 15 times the income of an African person. Health inequities, which are differences in health status between population groups that are socially produced, avoidable and unfair, are even larger than income inequities. A large body of evidence suggests a close relationship between income inequity and health inequity. This also relates to the large inequity in cancer prevention, care and treatment between and within high‐, middle‐, and low‐income countries. The high costs of innovative therapies will play a role in the sustainability of many healthcare systems.

Cancer care and treatment inequalities require scientific, public health policy, and moral considerations. Scientific and organizational innovations are emerging that may offer opportunities to reduce cancer‐related inequities. Recently, important science‐driven policy actions at scale have been taken, including the EU MoC, EBCP, the USA Cancer Moonshot by the National Cancer Institute, and various related programs in Asia and Africa. The new initiatives offer opportunities for reduced inequities when global cooperation is enhanced. Given the large burden of disease caused by cancer this opportunity must not be missed. Collaboration among governments, healthcare providers, non‐profit organizations, and community leaders is vital to address inequality in cancer care. Partnerships can help leverage resources, share best practices, and implement coordinated efforts to reach underserved populations. Cancer science itself should consider equity implications. International development funding should promote capacity building in cancer research and establishment of the necessary infrastructures, as well as provide incentives for innovation and collaboration.

Data sharing and science cooperation are essential to foster innovative research, including personalized/precision cancer medicine. International collaboration based on sharing patient data, biological materials, technological resources and competencies is necessary to optimize research for cancer prevention and therapeutics/care. Embracing technology and digital health solutions may help bridge the gap in cancer care. Telemedicine can provide remote access to cancer specialists, facilitate virtual consultations, and offer remote monitoring for patients in underserved areas.

Inequities resulting from the lack of affordability of cancer treatment and care must be addressed. To achieve this, low‐ and middle‐income countries require support for collaborative actions to build up locally adapted, dedicated cancer treatment centers with diagnostics, radiotherapy, surgery, and adequate access to a subset of cancer medicines with proven effectiveness. Improving access to quality healthcare services is crucial. This can be achieved by expanding healthcare coverage, reducing financial barriers of high treatment costs by insurance, improving patient transportation options, and increasing the availability of cancer screening programs.

Enhancing health literacy and raising awareness about cancer prevention, early detection, and treatment options is essential. Educating communities about risk factors, symptoms, and the importance of regular screenings can help reduce disparities by enabling early intervention.

Putting those on the margins of our societies at the center of our actions when advancing cancer research, care, and prevention should be a global priority. Creating a more equal and inclusive healthcare system will be possible by advocating for policies prioritizing equitable access to cancer care and treatment. Collaboration among science, civil society, and religious communities can make a difference in achieving that.

## 9 Conference statement

During the Conference, a **Statement** was formulated and agreed upon by all participants representing a more detailed summary of recommendations with a focus on inequalities:

### 9.1 Part I – General recommendation

**1. Cancer prevention and therapeutics/care show important disparities between and within countries, which must be addressed**. In the last few decades, the impressive development of basic and technological research has offered unexpected clinical/prevention research opportunities. Still, translating discoveries into cancer therapeutics/care and prevention is severely hindered by a lack of integration with clinical and prevention research necessary to develop personalized/precision cancer medicine for all. Health disparities require better understanding because they are associated with other economic gradients (e.g., access to information that decreases risk factors and foster behaviors to face risk awareness). If countermeasures are not taken, inequalities will further increase disparities in access to innovations in anticancer opportunities. For example, bringing new technologies for the early detection of cancer using biomarkers to low‐ and middle‐income countries is becoming increasingly important. Likewise, liquid biopsies should be used to monitor cancer progression and therapy efficacy.

### 9.2 Part II – Specific recommendations for policy makers

**2. Interactions among important policy actions**, including the EU Mission on Cancer, Europe's Beating Cancer Plan, the US Cancer Moonshot by the National Cancer Institute, and cancer‐related programs in the Global South, need strengthening to implement comprehensive translational cancer research. The initiatives by the USA and EU offer opportunities for cooperation at scale, and both should seek opportunities for global reach, including low‐ and middle‐income countries. Healthcare, however, is not a competence within the domain of the EU, so the European Commission and Member States should align their priorities and policies to ensure that health expectations are delivered. In contrast, health research is a shared competence of the EU and the Member States, with the European Research Area and comprehensive programs such as the Missions. Nevertheless, to reach the necessary critical mass, a landscape of inclusive international research collaborations must be developed, including sharing advanced infrastructures and patients' data. This might require revisiting the EU General Data Protection Regulation (GDPR). The cancer research and action communities worldwide must strengthen their science policy activities to inform decision‐makers and civil society of the benefits that international collaborative research will bring to well‐being and national economies.

Potential incentives and instruments to diminish inequalities through innovation may include:

- Fostering the use of ‘prizes’ oriented toward reducing inequalities (in addition to those based on scientific or technical merit) to complement traditional incentives for innovation;
- Regulatory frameworks that will encourage pharmaceutical firms to alleviate inequalities in access to drugs;
- Adopting ‘advanced market commitments’ at national and international levels (e.g., EU), under which governments commit to investing in translational research and/or guarantee reimbursement for a certain volume of a therapy that does not yet exist if market prospects are limited for some indications;
- Exploring alternative production methods and promoting local production for access to health technologies and medicines. Governments must support strategic projects to establish these.
- Promoting funding instruments and mechanisms which have appropriate representation of short‐term projects (e.g., ERC and similar institutions) and the long‐term programs typically needed for translational programs, for the different stages of the translational continuum from fundamental to implementation research, as well as for the different components of therapeutic/care research and prevention.
- Considering the potential impact of new technology on increasing cancer inequalities as the new technology is being developed, and taking effective measures to reduce the likelihood of this happening.

**3. Inequalities resulting from lack of affordability must be reduced and overcome**. Well‐equipped cancer treatment centers with expert personnel offering high‐quality multidisciplinary cancer care are prerequisites in all countries; this shall be the vision for all nations and peoples. However, low‐ and middle‐income countries require support for collaborative actions to reap substantial benefits from even relatively modest, dedicated cancer treatment centers with good diagnostics, radiotherapy, surgery, and adequate access to a subset of cancer medicines with proven effectiveness, which is likely easier to put in place. Complemented with legislation (including restricting access to tobacco; and differential patenting to avoid patent protection in those countries), public cancer awareness programs, screening for early detection and active prevention (HPV vaccination), this could lead to substantial improvements. Affordability plays a critical role, including information on clinical effectiveness, health economics and the pricing of drugs. International development funds should promote capacity building in cancer research and establishment of the necessary infrastructures, as well as provide incentives for innovation and collaboration. Moreover, new treatments, like chimeric antigen receptor (CAR) T cell therapy and bispecific antibodies, are changing the paradigm in hematologic malignancies, but inequities in access are immense. The high costs of these therapies will play a role in the sustainability of many healthcare systems. Therefore, efforts are needed to address and eliminate these disparities, especially for minorities and those in low‐ and middle‐income countries.

**4. Inequalities in cancer care and treatment require addressing ethical and moral issues**. The fight against poverty and increasing inequalities in access to cancer care and prevention deserve much more attention in terms of research and innovation efforts, coupled with funding and policies across the translational cancer research continuum. We must actively foster sustainable, healthy environments, not simply accept the implicit moral and ethical failures that result from these inequalities today. Putting those on the margins of our societies at the center of our actions when advancing cancer research and care/prevention should be a global priority. This requires international support for developing innovation ecosystems and policies integrating social needs, capacity building of national health systems and strengthening local research and industrial production capacity. From a humanitarian point of view, it is important to involve all cancer patients, with specific attention to the Global South (Africa, Asia, and South America), where poverty and population growth in the coming decades could aggravate unequal access to cancer care and prevention. From an innovation point of view, international collaboration based on sharing patients' data, biological materials, technological resources and competencies is necessary for optimizing research for prevention and therapeutics/care.

### 9.3 Part III – Specific recommendations for pharmaceutical industry

**5. The pharmaceutical and medical device industry is a critical partner in the cancer research continuum** and is pivotal in developing and testing new drugs and technologies. Pharmaceutical firms should play an active part in reducing inequalities to drug access, including pricing policy. In addition, academia and pharma have to take responsibility for a complete drug development process (which is not the case today), facilitate rapid testing of single agents, evaluate combination therapies, and find ways to secure patient affordability. Similar arguments apply to health technologies. Fair but societally acceptable returns are also motivated by the notion that innovations come from academia with support from public funding. The latter also holds for individual investigators starting up new companies. In the long run, revisiting patent laws to remove counterproductive incentives is desirable. These may include adopting regulatory frameworks based on ‘differential patenting’ for developing countries so that treatments would not be subject to patent protection in those countries. This also applies to the data industry, producers of medical devices and academia and the arrangements between them.

### 9.4 Part IV – Specific recommendations for health, academic and research leaders

**6. Basic biological and technological research drives innovative translational cancer research for therapeutics/care and prevention but must be better integrated with clinical research endeavors**. The research agenda should aim to decrease cancer incidence through prevention and by increasing cure rates through improved cancer screening and better treatment to reduce cancer deaths and avoid burdening healthcare systems by making cancer a chronic disease. Increasing attention should be paid to preclinical research to improve the coherence of the research continuum for translational cancer research. In addition, more interactions are needed between basic and clinical researchers to prioritize and prepare for the effective development of proof‐of‐concept clinical trials and prevention strategies. Translational research is bidirectional. With expanding technologies, extensive analyses are possible, e.g., fine‐needle and liquid biopsies or novel imaging techniques from patients during treatment, offering opportunities for bed‐to‐bench translational research. Further, the final segments of the drug development and medical device and technologies research, including implementation research and integration of health‐related quality of life research, need more support, as do outcomes and health economics research. The costs of cancer therapeutics/care are increasing due to treatment with expensive anticancer agents. Integration of outcomes and health economics research makes assessments of clinical effectiveness and cost‐effectiveness possible with tools for prioritization by the healthcare systems.

**7. Structuring translational and clinical cancer research nationally is a prerequisite for implementing personalized/precision cancer medicine and limiting inequalities within countries**. Translational research has to cover all cancer therapeutics/care and prevention components. Integration with healthcare is essential, and Comprehensive Cancer Centers (CCCs) should be responsible for orchestrating multidisciplinary cancer therapeutics/care, reaching out to areas with several million inhabitants. Quality of care and innovation through research are two sides of the same coin. Moving toward personalized/precision cancer medicine requires complex infrastructures for molecular pathology, genomics and advanced imaging enabling clinical trials with molecularly stratified patients. These infrastructures are only available in advanced cancer research centers, and today, most patients are diagnosed and treated outside such centers. New forms of collaboration with centralized molecular pathology directing the treatment of patients where they live can increase innovation and, at the same time, mitigate inequalities.

**8. Sharing technological resources and patient data will stimulate other research activities focusing on health‐related quality of life, like rehabilitation, psycho‐oncology, survivorship, and supportive and palliative care**. CCCs should establish clinical cancer registries for all their patients. This will enable outcomes research to assess the clinical effectiveness of therapeutics/care. Proper integration/exchange with national and international registries can enhance their utility. Health economics research to evaluate cost‐effectiveness based on outcomes data provides important information for prioritization. Furthermore, concerted actions and an open registry initiated by the Mission on Cancer and Europe's Beating Cancer Plan can pave the way for mitigating economic and social inequalities in low‐ and middle‐income countries with less‐developed health systems. In the long run, these efforts will also ensure that science‐driven and social innovations reach patients across the healthcare systems. They need to be complemented by the social appreciation of cancer research and care, and this requires further promoting science awareness and science education for all.

**9. Data sharing and critical mass are required for innovative research to develop personalized/precision cancer medicine. The amount of data will steadily increase, posing a challenge for preserving and sharing these in the future**. The number of patients needed, the biological diversity of tumors and normal samples, the fast‐rising amount of clinical and biological information, and the rapidly growing portfolio of medical and technological therapeutic approaches require increasingly sophisticated infrastructures and highly specialized staff to conduct research aimed at personalized/precision cancer medicine, including data handling and processing. The digitalization of the cancer research continuum is already becoming a reality, from digital pathology to digital outcome research. International collaboration based on sharing patient data and referring patients to specialized services with the necessary resources and technological competencies is required to optimize prevention and therapeutics/care research. In addition, promoting open access to new knowledge through ‘digital observatories’ of cancer research and care should be prioritized.

**10. Broadening the information base in line with populations' genomic diversity**. It is important to prioritize the inclusion of a wider diversity of genomes in genomic databases to translate precision medicine research into practice in different populations. Most studies contributing to this knowledge are based on populations of European ancestry, providing a reasonable genetic representation of individuals of European origin but a poor representation of other ethnic groups. The underrepresentation of non‐European populations in genomic databases is problematic because it may miss gene‐disease relationships for which the exposure or outcome is rare in European people. Furthermore, it limits the generalizability of findings from genomic research and its translation into clinical care in diverse populations.

**11. Involvement of patient representatives in structuring translational cancer research should have a high priority**. By definition, translational cancer research, a coherent cancer research continuum, is aimed at patients' health problems and individuals at risk. In addition, the patients' experiences are fundamental for cancer therapeutics/care, with health‐related quality of life as an important endpoint. With this background, patient representatives need to collaborate more directly with decision‐makers for cancer therapeutics/care and research. They are often participants in the leadership of funding agencies, CCCs and major research programs and involved in the prioritization, planning, and execution of research projects. EACS has a patient representative on the Board. The involvement of patients guarantees that high‐quality multidisciplinary cancer care is the goal of a CCC and that translational research, also for prevention, has a strong focus on prioritized research areas of relevance for patients and individuals at risk.

**12. Advanced education and research are key to increasing innovation and mitigating inequalities and demands for improved research career paths**. Education must promote the integration of basic, preclinical, and early clinical/prevention research to enhance the link between basic and clinical sciences. The education of young researchers should include translational research with a clear focus on patient needs. Attractive MD/PhD programs should be put in place. Exchange programs for young researchers should be further expanded, thereby contributing to the sustainability of CCCs, which will secure the education of the next‐generation of leaders. The success of international cooperation will depend on the career paths at the institutional level in each region/country. The latter will require improving recruitment, rewarding and assessment systems to increase the appreciation and value of research performance beyond scientometry, thus encouraging openness, humanism, collaboration and sharing to increase research quality and impact.

## 10 Conference participants

Joachim von BRAUN, President of the Pontifical Academy of Sciences, Bonn University.

Card. Peter K.A. TURKSON, Chancellor of the Pontifical Academy of Sciences.

Ulrik RINGBORG, EACS Secretary General, and Cancer Center Karolinska, Stockholm, Sweden.

Michael BAUMANN, EACS President, and German Cancer Research Center, Heidelberg, Germany.

Tit ALBREHT, Head of the Centre for Health Care, National Institute of Public Health, Ljubljana, Slovenia.

Anton BERNS, EACS and The Netherlands Cancer Institute, Amsterdam, The Netherlands.

Michael BOUTROS, EACS and German Cancer Research Center, Heidelberg, Germany.

Julio CELIS, EACS and Danish Cancer Institute, Copenhagen, Denmark.

Mammen CHANDY, Director, Tata Medical Center, Kolkata, Christian Medical College and Hospital, Vellore, India.

Chien‐Jen CHEN, PAS Academician, Academia Sinica, Taipei, Taiwan.

Alberto COSTA, EACS and European Commission, Cabinet of Commissioner Stella Kyriakides.

Francesco DE LORENZO, EACS and European Cancer Patient Coalition, Brussels, Belgium.

Edward DE ROBERTIS, PAS Academician, University of California, Los Angeles, USA.

Frederick Charles DUBEE, Senior Member of the BGI team, Finland.

Alexander EGGERMONT, EACS and University Medical Center Utrecht & Princess Máxima Center for Pediatric Oncology, Utrecht, The Netherlands.

Ingemar ERNBERG, Karolinska Institutet, Stockholm, Sweden.

Jesper FISKER, Association of European Cancer Leagues, Brussels, Belgium.

Mariya GABRIEL, European Commissioner for Innovation, Research, Culture, Education and Youth.

Edith HEARD, PAS Academician, European Molecular Biology Laboratory, Heidelberg, Germany.

Manuel HEITOR, EACS and University of Lisbon, Portugal.

Åslaug HELLAND, Oslo University Hospital, Norway.

Rui HENRIQUE, Porto Comprehensive Cancer Center, Portugal.

Andrés JATO, Swedish Ambassador to the Holy See.

Eva JOLLY, Karolinska Comprehensive Cancer Center, Stockholm, Sweden.

Bengt JÖNSSON, EACS and Stockholm School of Economics, Stockholm, Sweden.

Olli KALLIONIEMI, EACS and Science for Life Laboratory, Stockholm, Sweden.

Jan KORBEL, EACS and European Molecular Biology Laboratory, Heidelberg, Germany.

Mechthild KRAUSE, EACS and Carl Gustav Carus University Hospital, Dresden, Germany.

Douglas R. LOWY, National Institutes of Health, National Cancer Institute, USA.

Claudia MAYER, German Cancer Research Center, Heidelberg, Germany.

René MEDEMA, EACS and Director of Research at The Netherlands Cancer Institute, Amsterdam, The Netherlands.

Olivier MICHIELIN, CHUV Centre Hospitalier Universitaire Vaudois, Lausanne, Switzerland.

Peter NAGY, EACS and National Institute of Oncology, Budapest, Hungary.

Kristina NILSSON, Officer Embassy of Sweden to the Holy See.

Simon OBERST, Organisation of European Cancer Institutes, Brussels, Belgium.

H.E. Msgr. Vincenzo PAGLIA, President, Pontifical Academy for Life.

Vito PANSADORO, President, Vincenzo Pansadoro Foundation, for Uro‐Oncology Research, Director, Center for Laparoscopic and Robotic Urology, Rome, Italy.

M. Iqbal PARKER, University of Cape Town and African Academy of Sciences, South Africa.

Kevin RYAN, EACR, Cancer Research UK Scotland Institute, Glasgow, UK and Molecular Oncology Editorial Office, FEBS Press, Cambridge, UK.

Marcelo SÁNCHEZ SORONDO, Past Chancellor of the PAS.

Charles L. SAWYERS, Howard Hughes Medical Institute, Chevy Chase, MD, USA.

Joachim SCHÜZ, EACS and International Agency for Research on Cancer (IARC/WHO), Lyon, France.

Laurent SIMONS, University of Antwerp, Belgium.

Magdalena SKIPPER, Geneticist and the editor‐in‐chief, Nature.

Eric SOLARY, EACS and Gustave Roussy Cancer Campus Grand Paris, Villejuif, France.

David THOMAS, Garvan Institute of Medical Research, The Kinghorn Cancer Centre, Sydney, Australia.

Jan‐Willem VAN DE LOO, Policy Officer cancer research and innovation European, Commission, DG Research & Innovation.

Alexandra VALKENBURG, Head of EU Delegation to the Holy See.

Ingrid VAN DEN NEUCKER, EACS Executive Director Cancer Science Policy, Brussels, Belgium.

Christina VON GERTTEN, EACS Coordinator, Karolinska Institutet, Sweden.

Elisabete WEIDERPASS, EACS and International Agency for Research on Cancer (IARC/WHO), Lyon, France.

Huanming YANG, Beijing Genomics Institute (BGI), Shenzhen, China.

## Conflict of interest

The authors declare no conflict of interest.
